# Supplementary material for: Conversion of La2Ti2O7 to LaTiO2N via ammonolysis: a first-principles investigation
Source: Phys Chem Chem Phys. 2023 Jul 21;25(30):20575–84. doi: 10.1039/d3cp02159a (PMC10395003; doi:10.1039/d3cp02159a)
Supplement: CP-025-D3CP02159A-s001 [file CP-025-D3CP02159A-s001.pdf]

Electronic Supplementary Material (ESI) for Physical Chemistry Chemical Physics.
This journal is © the Owner Societies 2023

Supplementary information for

Conversion of $La_2Ti_2O_7$ to $LaTiO_2N$ via Ammonolysis: A First-Principles Investigation

Chiara Ricca,[1,2] Tristan Blandenier,[1] Valérie Werner,[3] Xing Wang,[1] Simone Pokrant[3] and Ulrich Aschauer[1,2,3*]
[1]*Department of Chemistry and Biochemistry, University of Bern, Freiestrasse 3, CH-3012 Bern, Switzerland*
[2]*National Centre for Computational Design and Discovery of Novel Materials (MARVEL), Switzerland*
[3]*Department of Chemistry and Physics of Materials, University of Salzburg, Jakob-Haringer-Str. 2A, A-5020 Salzburg, Austria*

Email: ulrich.aschauer@plus.ac.at

(Dated: June 26, 2023)

CONTENTS

S1. Stoichiometric bulk materials S2

S2. Phase Diagrams S3
A. LTO S3
B. LTON S3

S3. Interaction of LTO (001) with NH_3 S5

S4. Defect Chemistry of LTO S6
A. Oxygen vacancies (V_O) S6
B. Substitutional nitrogen (N_O) S9
C. Interstitial nitrogen (N_i) S11
D. Substitutional N and interstitial O ($N_O{-}O_i$) S13
E. Substitutional N and oxygen vacancies ($N_O{-}V_O$) S15
F. Interstitial N and substitutional N ($N_i{-}N_O$) S17
G. Two substitutional N ($N_O{-}N_O$) S20
H. N diffusion S22

S5. Defect Chemistry of LTON S23
A. Oxygen (V_O) and nitrogen (V_N) vacancies S23
B. Substitutional nitrogen (N_O) and oxygen (O_N) S25
C. Interstitial nitrogen (N_i) and oxygen (O_i) S27
D. Interstitial nitrogen and oxygen vacancy ($N_i{-}V_O$) S31
E. Interstitial oxygen and oxygen vacancy ($O_i{-}V_O$) S33
F. La_{Ti} cation antisites and complexes S35
G. Ti_{La} cation antisites and complexes S37

S6. Effect of the N chemical potential S39

References S41

S1. STOICHIOMETRIC BULK MATERIALS

The lattice parameters of LTO computed at the PBE+U level of theory are a=7.774 Å, b=5.537 Å, c=13.165 Å, β=81.5°, in good agreement with experimental values[1] (relative error of about 1%). The electronic density of states of pristine LTO is reported in Fig. S1: the valence band (VB) is dominated by O-2p states with smaller contributions of the Ti-3d and La orbitals, reflecting the partially covalent character of the Ti−O bonds, while the bottom of the conduction band (CB) has mainly Ti-3d character. The computed band gap of 3.05 eV slightly underestimates experiment (3.29-3.82 eV)[2–4], due to the limited effect of the Hubbard correction of the empty Ti-3d states for a material with nominal d^0 transition-metal configuration.

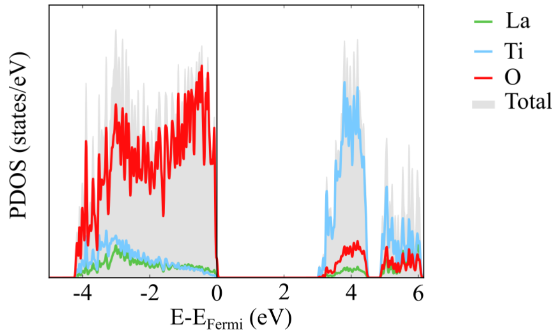

FIG. S1: Total and atom-projected density of states (PDOS) for LTO.

For LTON we considered a model with *cis* order of the N atoms on the anion sublattice. In Fig. S2 we confirm the previously reported small N-2p shoulder at the top of the valence band, with Ti-3d orbitals forming the bottom of the conduction band[5]. The computed band-gap of 1.43 eV, is inevitably smaller than the experimental value of 2.10 eV[?] within our GGA+U framework.

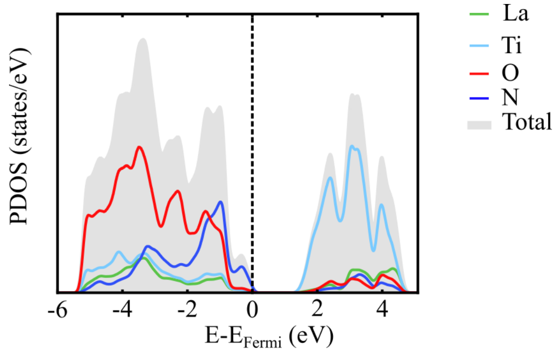

FIG. S2: Total and atom-projected density of states (PDOS) for LTON.

S2. PHASE DIAGRAMS

A. LTO

The LTO phase diagram in Fig. S3 was derived considering that, at equilibrium, the chemical potentials should satisfy

$$2\mu_{\mathrm{La}} + 2\mu_{\mathrm{Ti}} + 7\mu_{\mathrm{O}} = \Delta H_f(\mathrm{LTO}), \tag{S1}$$

where ΔH_f(LTO) is the heat of formation of bulk LTO. Since the O chemical potential is controlled during the transformation of LTO to LTON by the ammonia flow removing oxygen, different synthesis conditions are considered by adjusting the oxygen chemical potential ($\mu_{\mathrm{O}} = \mu_{\mathrm{O}}^0 + \Delta\mu_{\mathrm{O}}$). We assume equilibrium with O_2 gas to determine $\mu_{\mathrm{O}}^0 = \frac{1}{2}\mu_{\mathrm{O}_2}$, where μ_{O_2} is the computed total energy of the O_2 molecule. $\Delta\mu_{\mathrm{O}}$ can then vary within a range limited by the formation of competing phases like La_2O_3 ($2\mu_{\mathrm{La}} + 3\mu_{\mathrm{O}} \leq \Delta H_f(\mathrm{La_2O_3})$) and TiO_2 ($\mu_{\mathrm{Ti}} + 2\mu_{\mathrm{O}} \leq \Delta H_f(\mathrm{TiO_2})$) and by the decomposition of LTO into metallic La ($\mu_{\mathrm{La}} < \mu_{\mathrm{La,metal}}$) and Ti ($\mu_{\mathrm{Ti}} < \mu_{\mathrm{Ti,metal}}$). The DFT+$U$ computed formation energies, derived according to Ref. 6, are in good agreement with those from experiment (Table S1). We will report defect formation energies in LTO for O-poor conditions ($\Delta\mu_{\mathrm{O}} = -4.54$ eV) relevant for ammonolysis.

TABLE S1: Comparison of DFT+U computed and experimental formation energies (eV/formula unit).

Material	DFT+U	Expt.[7]
La_2O_3	-17.18	-18.59
TiO_2 (rutile)	-9.07	-9.74
LTO	-36.70	-40.33
LTON	-13.43	-14.69

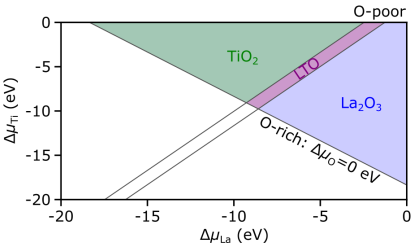

FIG. S3: Computed phase diagram for LTO. LTO is stable in the purple region, while in the green and blue shaded areas TiO_2 and La_2O_3 respectively form. Also indicated is the O-poor compositional limit as well as the O-rich limit.

B. LTON

The LTON phase diagram in Fig. S4 was derived considering that, at equilibrium, the chemical potentials should satisfy

$$\mu_{\mathrm{La}} + \mu_{\mathrm{Ti}} + 2\mu_{\mathrm{O}} + \mu_{\mathrm{N}} = \Delta H_f(\mathrm{LTON}), \tag{S2}$$

where ΔH_f(LTON) is the heat of formation of bulk LTON. Only N-rich conditions ($\Delta\mu_{\mathrm{N}} = 0$ eV) were considered because the environment is saturated with NH_3 during thermal ammonolysis. The O chemical potential was limited by the same competing phases considered above for LTO and in addition also the formation of LTO as a competing phase ($2\mu_{\mathrm{La}} + 2\mu_{\mathrm{Ti}} + 7\mu_{\mathrm{O}} = \Delta H_f(\mathrm{LTO})$). We will report defect formation energies in LTON for O-poor conditions ($\Delta\mu_{\mathrm{O}} = -6.72$ eV) relevant for ammonolysis.

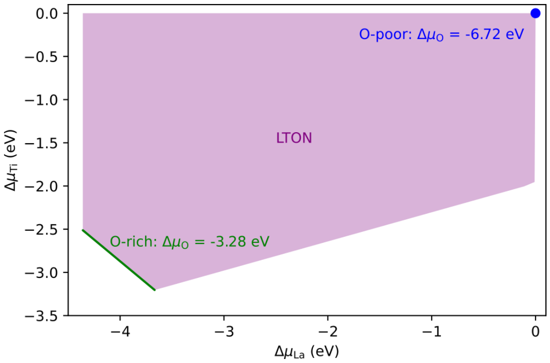

FIG. S4: Computed phase diagram for LTON for $\Delta\mu_{\mathrm{N}} = 0$ eV, corresponding to N-rich conditions. LTON is stable in the purple region. The green line and the blue dot represent O-rich and O-poor conditions, respectively.

To ease comparison with experiment, the chemical potentials ($\Delta\mu_{\mathrm{O}}$) were converted to oxygen partial pressure via

$$\mu_0 + \Delta\mu = \mu = \mu_0 + kT \ln\left(\frac{p}{p_0}\right) \Rightarrow p = p_0 \cdot \exp\left(\frac{\Delta\mu}{kT}\right) \tag{S3}$$

where p_0 is the standard pressure of $21\% \cdot 1$ bar, k the Boltzmann constant and we chose a typically ammonolysis temperature of $T = 1223$ K.

S3. INTERACTION OF LTO (001) WITH NH_3

Table S2 reports the relative energies of the different oxygen vacancy sites at the LTO (001) surface. Figure S5 shows top views of the stoichiometric surface as well as the two most stable oxygen vacancy sites, both of which are located at the very surface on top of a Ti atom. In the remaining calculations we considered V_O-41 only.

TABLE S2: Relative energies of the defective slabs for different oxygen vacancy sites.

V_O site	Relative energy (eV)
V_O-30	0.01
V_O-31	0.29
V_O-35	0.11
V_O-37	0.18
V_O-40	0.09
V_O-41	0.00

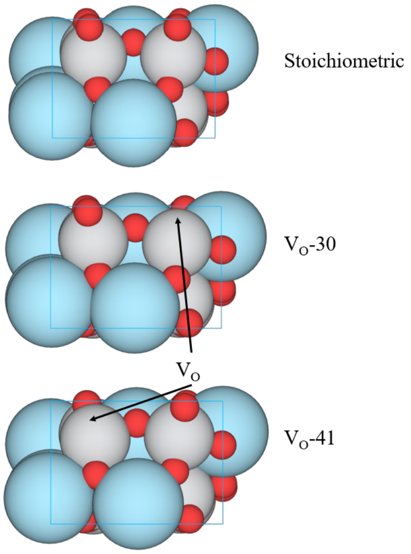

FIG. S5: Top view of the stoichiometric slab as well as the two slabs with oxygen vacancies at the most stable sites.

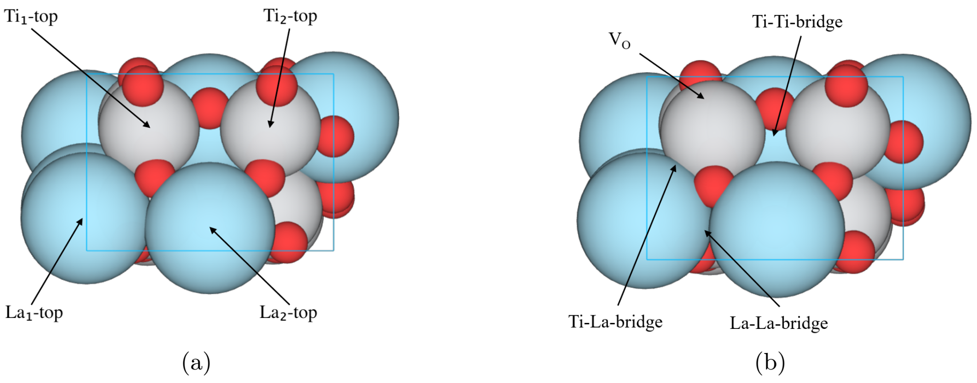

FIG. S6: Adsorption sites on (a) the stoichiometric and (b) the defective surface.

S4. DEFECT CHEMISTRY OF LTO

A. Oxygen vacancies (V_O)

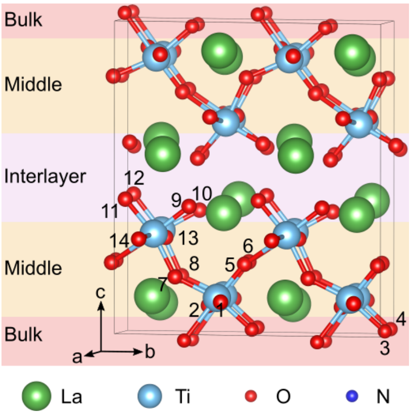

FIG. S7: $1 \times 2 \times 1$ LTO supercell. Purple, orange, and red shaded areas correspond to the interlayer, the middle or bulk layer respectively. The numbers indicate the 14 different sites from which O atoms were removed when forming V_O or N_O.

We considered doubly and singly positively charged as well as neutral oxygen vacancies in LTO. Neutral ${V_O}^0$ are associated with the two extra electrons in a shallow defect state dominated by Ti-3d orbitals and centered on the vacancy site (Fig. S8a and d). For a singly charged ${V_O}^{+1}$, a singly occupied defect state much deeper in the band gap leads to reduction of one of the Ti atoms adjacent to the defect (Fig. S8b and e). Finally, an empty defect appears at the bottom of the CB for doubly charged ${V_O}^{+2}$ (Fig. S8c and f). Due to the low symmetry of monoclinic LTO, there are 14 different sites for V_O as shown in Fig. S7.

Figure S9 reports the formation energy computed in the O-poor limit for the different configurations and charge states. Increasing the defect charge stabilizes configurations lying in the interlayer and destabilizes those in the middle and bulk layer (Table S3). The large difference in formation energy for V_O within the same layer is due to the symmetry lowering octahedral rotations, as revealed by comparing Fig. S9a with data obtained for neutral defects in a cell without rotations (Fig. S11).

The change in V_O formation energy as a function of the Fermi energy (E_{Fermi}) indicates a transition of the most stable charge state from ${V_O}^{+2}$ to ${V_O}^{+1}$ at a Fermi energy of about 2.2 eV, and finally to ${V_O}^0$ for E_{Fermi} close to the CBM (Fig. S10a). Under experimentally relevant conditions, we expect to find neutral ${V_O}^0$ in sites further than 1.9 Å from the interlayer interface. The formation energy for this defect increases from about 1 eV in O-poor to 6 eV in O-rich conditions as shown in Fig. S10b), suggesting that the oxygen-vacancy concentration may not be very high. This is in line with the experimental evidence that V_O are important to trigger the zipper mechanism and that the nitridation gas-diffusion path can be quite long before a defect able to start the chain reaction is encountered.

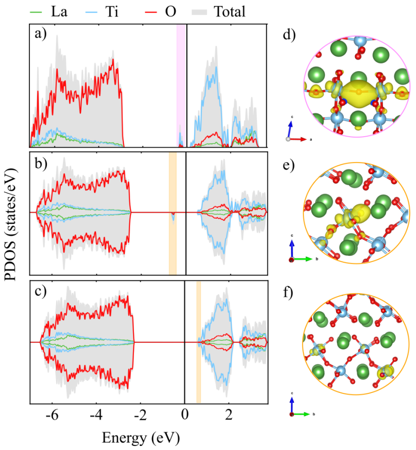

FIG. S8: Total and atom-projected density of states (PDOS) for a) $V_O{}^0$, b) $V_O{}^{+1}$, and c) $V_O{}^{+2}$ in LTO. For each defect, the zero of the energy scale was set at the respective Fermi energy. The 10^{-2} e/Å isosurfaces in panels e-f show the density associated with the defect states (energy range highlighted in the PDOS).

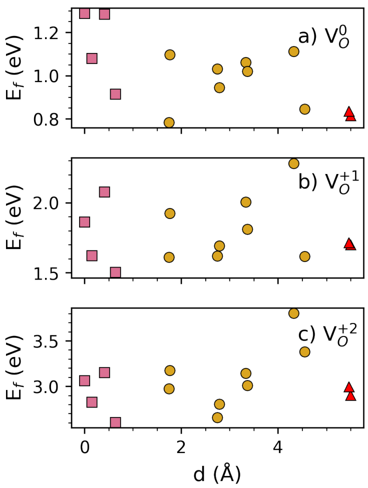

FIG. S9: Formation energy of the different configurations for a) $V_O{}^0$, b) $V_O{}^{+1}$, and c) $V_O{}^{+2}$ in LTO as a function of the distance of the defect from the interlayer interface. Pink squares, golden circles, and red triangle correspond to defects lying in the interlayer, middle, or bulk layers as depicted in Fig. S7.

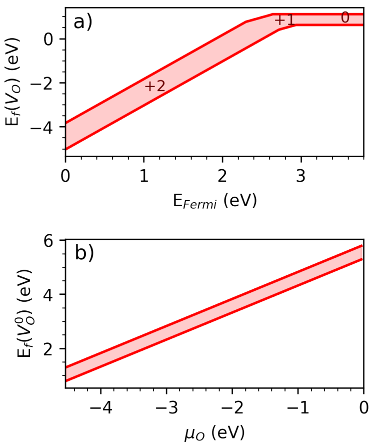

FIG. S10: a) V_O formation energy ($E_f(V_O)$) in different charge states computed as a function of the Fermi energy (E_{Fermi}, up to the experimental band gap E_g^{expt}) referenced to the valence band maximum of LTO and in the O-poor limit ($\Delta\mu_O$=-4.54 eV). Only the most stable charge state is reported and indicated in each region. b) Formation energy of a neutral V_O as a function of the oxygen chemical potential and for $E_{Fermi} = E_g^{expt}$. The colored area indicates the variation of $E_f(V_O)$ for different sites.

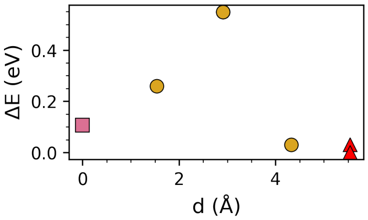

FIG. S11: Relative energy of the different configurations for a $V_O{}^0$ in a LTO cell without octahedral rotations. Pink squares, golden circles, and red triangle correspond to defects lying in the interlayer, middle, or bulk layers as depicted in Fig. S7.

TABLE S3: Formation energy (in eV) for a neutral $V_O{}^0$, singly positively $V_O{}^{+1}$, and doubly positively charged $V_O{}^{+2}$ oxygen vacancy computed for the 14 inequivalent O sites in the interlayer (I), middle (M), or bulk (B) layer of LTO (see Fig. S7 for site labels). Relative energies of a neutral $V_O{}^0$ in a cell without octahedral rotations (no rot.) are also reported.

Layer	Site	$V_O{}^0$	$V_O{}^0$ (no rot.)	$V_O{}^{+1}$	$V_O{}^{+2}$
I	11	1.08	0.11	1.62	2.82
I	10	0.92	0.11	1.50	2.60
I	12	1.29	0.11	1.86	3.06
I	09	1.28	0.11	2.08	3.15
M	02	0.85	0.03	1.62	3.38
M	13	0.78	0.26	1.61	2.97
M	01	1.11	0.03	2.28	3.80
M	06	1.03	0.55	1.62	2.66
M	07	1.06	0.55	2.00	3.14
M	05	0.95	0.55	1.69	2.81
M	08	1.02	0.55	1.81	3.01
M	14	1.10	0.26	1.92	3.17
B	03	0.81	0.03	1.70	2.90
B	04	0.84	0.00	1.72	3.00

B. Substitutional nitrogen (N_O)

As N has one less electron compared to O, when a neutral O atom is substituted by a neutral N, a neutral ${N_O}^0$ is formed and both filled and empty defect states appear below and above the Fermi level, which are respectively dominated by N-2p and O-2p orbitals (Fig. S12c). These impurity states were previously associated with the band gap reduction in N-doped LTO[8–10], but they could also act as recombination centers. More importantly, this type of doping would result in p-type conductivity, which is very unusual for a wide band gap metal oxide semiconductor, since acceptor states above the Fermi level are generally thermodynamically unstable. However, the change in formation energy of N_O as a function of the Fermi energy reported in Fig. S14a clearly indicates that ${N_O}^0$ is not a stable charge state. ${N_O}^{+1}$ is the most stable charge for E_{Fermi} lower than about 1 eV. The formation of the electron-deficient ${N_O}^{+1}$ is accompanied either by the appearance of an empty defect state at the top of the VB (Fig. S12a) or with the formation of a N−O bond when N substitutes for an O atoms close to the interlayer where the neighboring O atoms have enough flexibility to relax toward the substitutional N and stabilize it (Fig. S12e). For higher Fermi energies, i.e. under experimentally relevant conditions, ${N_O}^{-1}$ is the most stable charge state and is associated with fully occupied defect states merged with the VB, resulting in a band-gap reduction of about 0.6 eV, in good agreement with experimental observations for N-doped LTO[11]. In the experimentally relevant -1 charge state, N_O prefer sites in the middle layer (Fig. S13c). Formation energies for the different configurations are reported in Table S4.

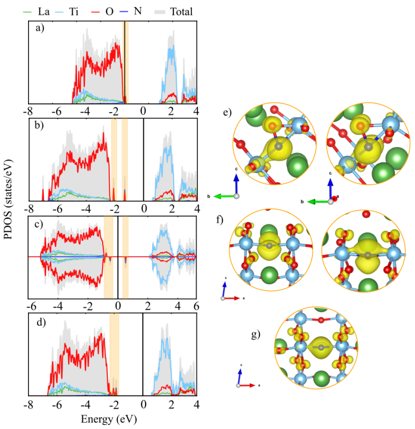

FIG. S12: Total and atom-projected density of states (PDOS) for one a) ${N_O}^{+1}$, b) ${N_O}^{+1}$ where N bonds to one O atom, c) ${N_O}^0$, d) ${N_O}^{-1}$. For each defect, the origin of the energy scale was set at the respective Fermi energy. The 10^{-2} e/Å isosurfaces show the density associated with the defect states (energy range highlighted in the PDOS.

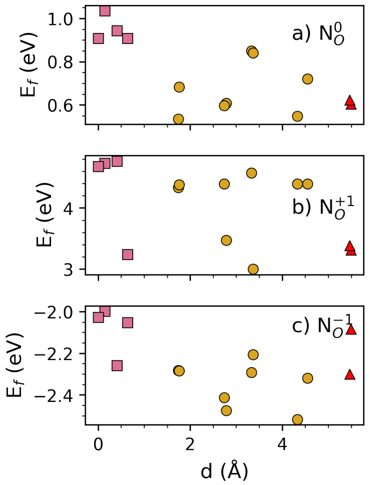

FIG. S13: Formation energy of the different configurations for a) ${N_O}^0$, b) ${N_O}^{+1}$, and c) ${N_O}^{-1}$ in LTO as a function of the distance of the defect from the interlayer interface. Pink squares, golden circles, and red triangle correspond to defects lying in the interlayer, middle, or bulk layers as depicted in Fig. S7.

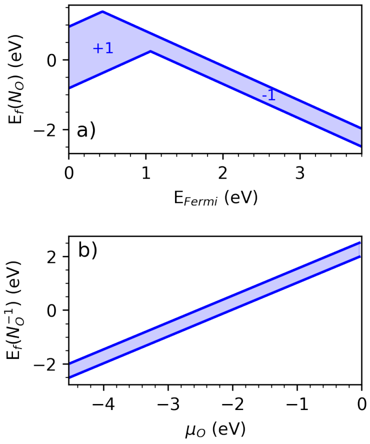

FIG. S14: a) Nitrogen substitutional formation energy ($E_f(\mathrm{N_O})$) in different charge states computed as a function of the Fermi energy (E_{Fermi}, up to the experimental band gap E_g^{expt}) referenced to the valence band maximum of LTO and in the O-poor limit ($\Delta\mu_O$=-4.54 eV). Only the most stable charge state is reported and indicated in each region. b) Formation energy for a ${N_O}^{-1}$ as a function of the oxygen chemical potential and for $E_{\mathrm{Fermi}} = E_g^{\mathrm{expt}}$. The colored area indicates the variation of $E_f(\mathrm{N_O})$ for different sites.

TABLE S4: Formation energy (in eV) for a neutral ${N_O}^0$, singly positively ${N_O}^{+1}$, and singly negatively charged ${N_O}^{-1}$ substitutional nitrogen at an oxygen site computed for the 14 inequivalent O sites in the interlayer (I), middle (M), or bulk (B) layer of LTO (see Fig. S7 for site labels).

Layer	Site	${N_O}^0$	${N_O}^{+1}$	${N_O}^{-1}$
I	11	1.03	4.73	-2.00
I	10	0.91	3.24	-2.05
I	12	0.91	4.68	-2.03
I	09	0.94	4.77	-2.26
M	02	0.72	4.40	-2.32
M	13	0.54	4.34	-2.28
M	01	0.55	4.39	-2.52
M	06	0.60	4.39	-2.41
M	07	0.85	4.57	-2.29
M	05	0.61	3.48	-2.48
M	08	0.84	3.00	-2.21
M	14	0.68	4.38	-2.28
B	03	0.60	3.31	-2.08
B	04	0.62	3.38	-2.30

C. Interstitial nitrogen (N_i)

The most stable charge state for an interstitial N is ${N_i}^0$ for Fermi energies below 2 eV, ${N_i}^{-1}$ between 2 and 2.7 eV, and finally ${N_i}^{-3}$ for Fermi energies close to the experimental band gap (Fig. S17a). Interestingly, regardless the charge state, strong structural relaxations are observed with N_i migrating towards one of the neighboring O atoms and finally resulting in a substitutional nitrogen at an O site and an interstitial O bonded to N. This results in N-2p and O-2p states appearing at the top and bottom of the VB (Fig. S15). This result is line with XPS spectra of N-doped LTO that show the environment of La not to be affected by the doping while N is bonded to Ti atoms as part of the Ti octahedra[11,12]. N_i show a slight preference for sites in the middle layer (Fig. S16) and a -3 charge state (Fig. S17). Details on the configurations can be found in Fig. S16 and Table S5, while the sites are shown in Fig. S18.

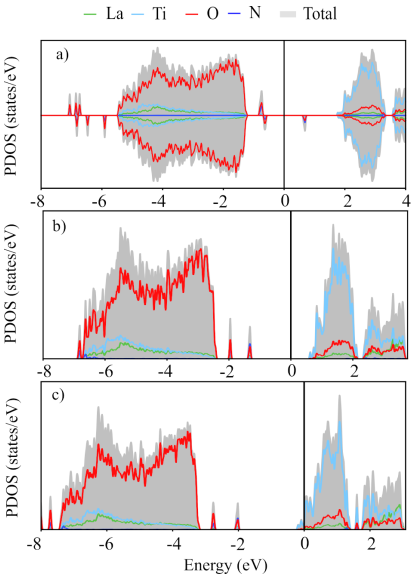

FIG. S15: Total and atom-projected density of states (PDOS) for a) ${N_i}^0$, b) ${N_i}^{-1}$, and c) ${N_i}^{-3}$. For each defect, the origin of the energy scale was set at the respective Fermi energy.

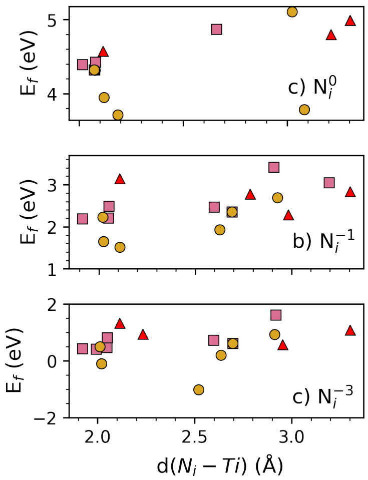

FIG. S16: Formation energy of the different configurations for a) ${N_i}^0$, b) ${N_i}^{-1}$, and c) ${N_i}^{-3}$ in LTO as a function of the distance between N_i and the closest Ti atom. Pink squares, golden circles, and red triangle correspond to defects in the interlayer, middle, or bulk layers as depicted in Fig. S7.

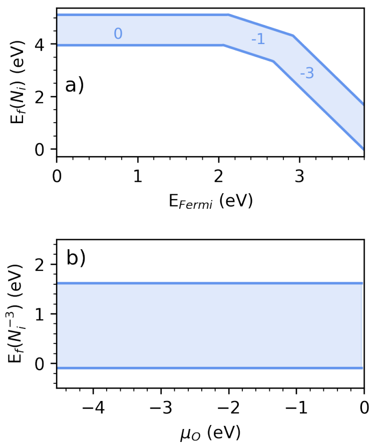

FIG. S17: a) Interstitial nitrogen formation energy ($E_f(\mathrm{N_i})$) in different charge states computed as a function of the Fermi energy (E_{Fermi}, up to the experimental band gap E_g^{expt}) referenced to the valence band maximum of LTO and in the O-poor limit ($\Delta\mu_{\mathrm{O}}$=-4.54 eV). Only the most stable charge state is reported and indicated in each region of the plot. b) Formation energy for a ${N_i}^{-3}$ as a function of the oxygen chemical potential and for $E_{\mathrm{Fermi}} = E_g^{\mathrm{expt}}$. In both cases, the colored area indicates the variation of $E_{\mathrm{f}}(\mathrm{N_i})$ for different sites.

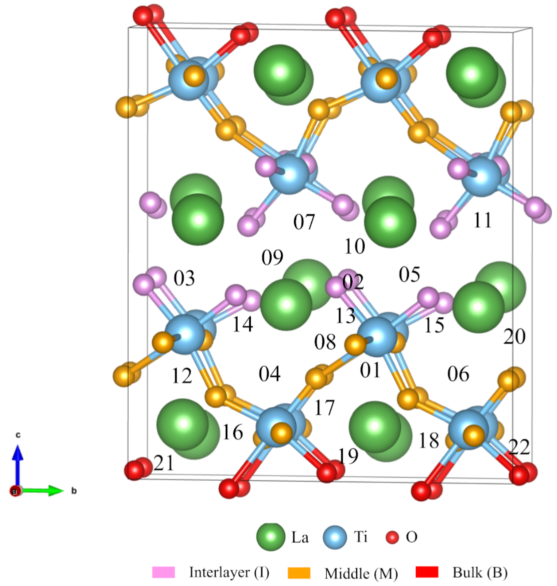

FIG. S18: $1 \times 2 \times 1$ supercell used for modeling intersititial nitrogen in LTO. Oxygen atoms colored in pink, orange, and red correspond to O lying in the interlayer region, in the middle and bulk layer. The numbers indicate the 22 sampled N_i interstitial sites.

TABLE S5: Formation energy (in eV) for a neutral ($N_i{}^0$), singly positively ($N_i{}^{+1}$), doubly positively ($N_i{}^{+2}$), singly negatively ($N_i{}^{-1}$) and triply negatively charged ($N_i{}^{-3}$) nitrogen interstitial computed for inequivalent N_i sites lying in the interlayer (I), middle (M), or bulk (B) layer of a LTO slab (cf. Fig. S18 for site labels).

Layer	Site	$N_i{}^0$	$N_i{}^{-1}$	$N_i{}^{-3}$
I	02	1.00	2.00	2.00
I	03	2.00	2.00	2.00
I	13	1.00	2.00	2.00
I	15	2.00	2.00	0.00
I	07	2.00	2.00	2.00
I	10	1.00	2.00	2.00
I	09	1.00	1.00	1.00
I	11	1.00	1.00	3.00
I	05	1.00	1.00	1.00
I	14	2.00	2.00	2.00
M	12	2.00	2.00	2.00
M	06	4.00	3.00	3.00
M	17	2.00	2.00	2.00
M	04	4.00	3.00	3.00
M	16	2.00	2.00	2.00
M	08	2.00	2.00	2.00
M	01	2.00	2.00	2.00
M	20	1.00	2.00	2.00
B	19	2.00	1.00	2.00
B	18	2.00	2.00	2.00
B	21	2.00	2.00	2.00
B	22	2.00	2.00	2.00

D. Substitutional N and interstitial O (N_O-O_i)

To further investigate the role of N_O-O_i, we studied this defect complex more in detail by fixing one N_O in the most stable position and by positioning the interstitial O atom at different sites in the LTO slab. The most stable charge state for Fermi energies close to the LTO band gap is $(N_O-O_i)^{-3}$ (Fig. S20a). In this charge state the two defects can be spatially separated, while for all other charge states, configurations with formation of a N_O-O_i bond are favored, larger N_O-O_i distances corresponding to higher formation energies (Fig. S19). Details on the configurations can be found in Fig. S21 and Table S5.

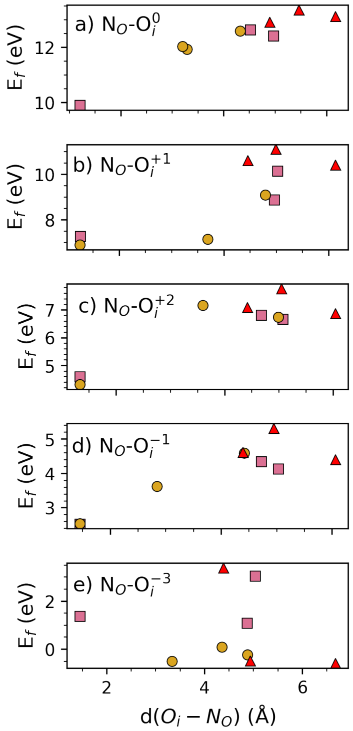

FIG. S19: Formation energy of the different configurations for a) $(N_O-O_i)^0$, b) $(N_O-O_i)^{+1}$, c) $(N_O-O_i)^{+2}$, d) $(N_O-O_i)^{-1}$, and e) $(N_O-O_i)^{-3}$ defect in LTO as a function of the distance between O_i and N_O. Pink squares, golden circles, and red triangle correspond to O_i lying in the interlayer, middle, or bulk layer as depicted in Fig. S21.

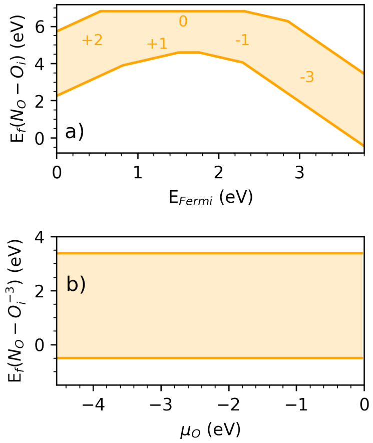

FIG. S20: a) N_O-O_i formation energy ($E_f(N_O-O_i)$) in different charge states computed as a function of the Fermi energy (E_{Fermi}, up to the experimental band gap E_g^{expt}) referenced to the valence band maximum of LTO and in the O-poor limit ($\Delta\mu_O$=-4.54 eV). Only the most stable charge state is reported and indicated in each region of the plot. b) Formation energy for a $(N_O-O_i)^{-3}$ as a function of the oxygen chemical potential and for $E_{Fermi}=E_g^{expt}$. In both cases, the colored area indicates the variation of $E_f(N_O-O_i)$ for different configurations.

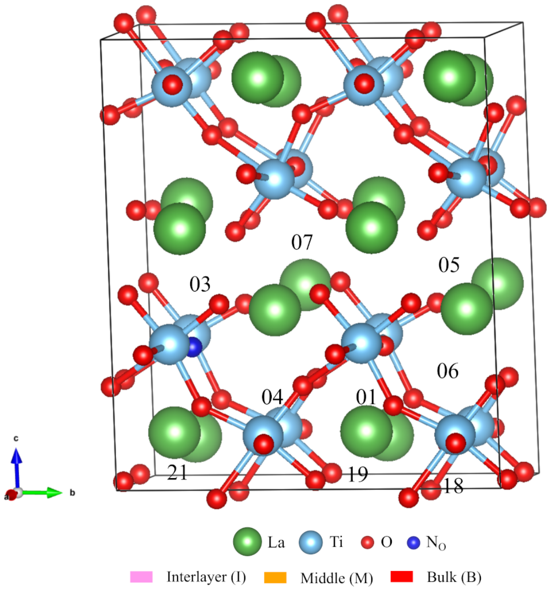

FIG. S21: $1 \times 2 \times 1$ supercell used for modeling $N_O{-}O_i$ in LTO. Oxygen atoms colored in pink, orange, and red correspond to O in the interlayer, the middle or bulk layer. The numbers indicate the different sites for an interstitial oxygen O_i while the substitutional nitrogen (N_O) located at the most stable position is shown in blue.

TABLE S6: Formation energy (in eV) for a neutral $(N_O{-}N_i)^0$, singly positively $(N_O{-}N_i)^{+1}$, doubly positively $(N_O{-}N_i)^{+2}$, singly negatively $(N_O{-}N_i)^{-1}$ and triply negatively charged $(N_O{-}N_i)^{-3}$ substitutional nitrogen - interstitial oxygen complex computed for inequivalent O_i sites in the interlayer (I), middle (M), or bulk (B) layer of LTO (see Fig. S21 for site labels).

Layer	Site	$N_O{-}N_i{}^0$	$N_O{-}N_i{}^{+1}$	$N_O{-}N_i{}^{-1}$	$N_O{-}N_i{}^{+2}$	$N_O{-}N_i{}^{-3}$
I	03	4.58	7.26	2.53	9.89	1.36
I	07	6.81	8.88	4.33	12.63	1.09
I	05	6.67	10.14	4.13	12.42	3.04
M	01	7.16	9.08	4.59	12.59	0.08
M	06	6.74	7.15	3.62	12.04	-0.24
M	04	4.32	6.90	2.54	11.93	-0.51
B	19	6.88	10.40	4.40	13.12	-0.60
B	18	7.76	11.10	5.31	13.36	-0.48
B	21	7.10	10.61	4.62	12.91	3.38

E. Substitutional N and oxygen vacancies (N_O-V_O)

We also considered the defect complex of a substitutional nitrogen and an oxygen vacancy (N_O-V_O) created by fixing the V_O close to the interlayer interface while positioning N_O at different distances from V_O. Results indicated that there is no general correlation between the N_O-V_O distance and the relative stability of the configuration, but they rather point towards the already known preference of N_O to lie in the middle layer (Fig. S23). Details on configurations can be found in Fig. S25 and Table S7. This defect in its experimentally most relevant -3 charge state (Fig. S24) induces defect states below the CB (Fig. S22).

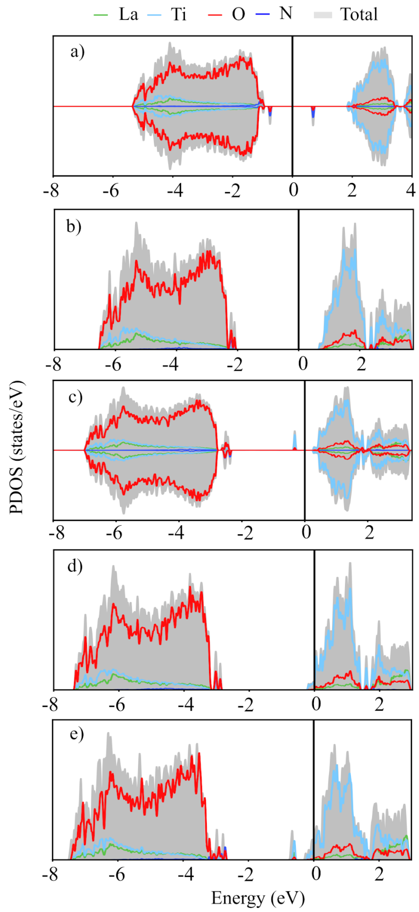

FIG. S22: Total and atom-projected density of states (PDOS) for a) $(N_O-V_O)^{+2}$, b) $(N_O-V_O)^{+1}$, c) $(N_O-V_O)^0$, d) $(N_O-V_O)^{-1}$, and e) $(N_O-V_O)^{-3}$. For each defect, the origin of the energy scale was set to the respective Fermi energy.

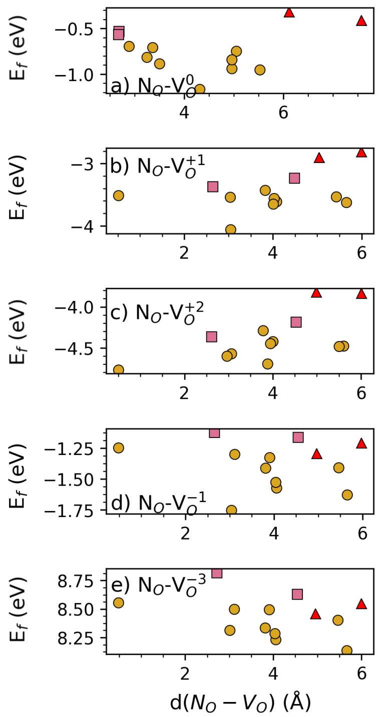

FIG. S23: Formation energy of the different configurations for a) $(N_O-V_O)^0$, b) $(N_i-V_O)^{+1}$, c) $(N_i-V_O)^{+2}$, d) $(N_i-V_O)^{-1}$, and e) $(N_O-V_O)^{-3}$ in LTO as a function of the distance between N_O and V_O. Pink squares, golden circles, and red triangle correspond to N_O lying in the interlayer, middle, or bulk layer as depicted in Fig. S25.

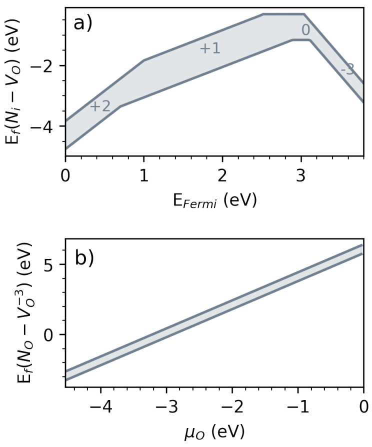

FIG. S24: a) N_O-V_O formation energy ($E_f(N_O - V_O)$) in different charge states computed as a function of the Fermi energy (E_{Fermi}, up to the experimental band gap E_g^{expt}) referenced to the valence band maximum of LTO and in the O-poor limit ($\Delta\mu_O$=-4.54 eV). Only the most stable charge state is reported and indicated in each region of the plot. b) Formation energy for a $(N_O-V_O)^{-3}$ as a function of the oxygen chemical potential and for $E_{Fermi} = E_g^{expt}$. The colored areas indicates the variation of $E_f(N_O - V_O)$ for different configurations.

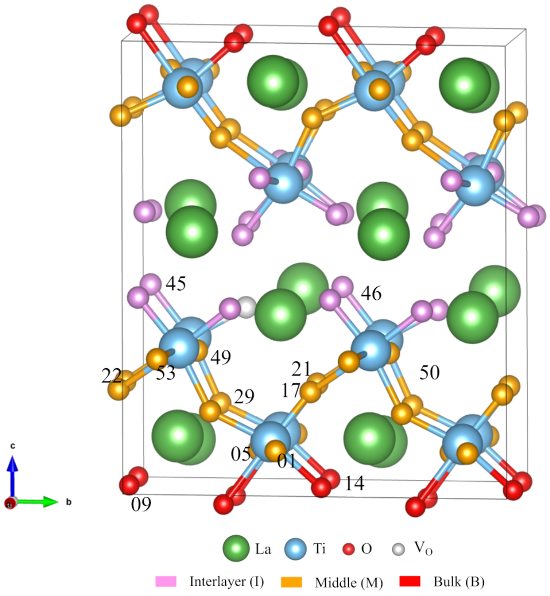

FIG. S25: $1 \times 2 \times 1$ supercell used for modeling substitutional N-oxygen vacancy pairs in LTO. Oxygen atoms colored in pink, orange, and red correspond to O lying in the interlayer region, in the middle or at bulk layer. The numbers indicate the different sites for the substitutional nitrogen N_O with the oxygen vacancy (V_O) fixed at the most stable position.

TABLE S7: Formation energy (in eV) for a neutral $(N_O-V_O)^0$, triply negatively $(N_O-V_O)^{-3}$, singly negatively $(N_O-V_O)^{-1}$, singly positively $(N_O-V_O)^{+1}$ and doubly positively charged $(N_O-V_O)^{+2}$ nitrogen substitutional - oxygen vacancy complexes computed for inequivalent N_O sites lying in the interlayer (I), middle (M), or bulk (B) layer (see Fig. S25 for site labels).

Layer	Site	$N_O-V_O{}^0$	$N_O-V_O{}^{-3}$	$N_O-V_O{}^{-1}$	$N_O-V_O{}^{+1}$	$N_O-V_O{}^{+2}$
I	46	-0.56	8.63	-1.16	-3.23	-4.19
I	45	-0.53	8.82	-1.12	-3.37	-4.36
M	01	-0.94	8.14	-1.63	-3.62	-4.48
M	21	-0.88	8.29	-1.52	-3.56	-4.45
M	22	-1.17	8.31	-1.75	-4.06	-4.57
M	05	-0.75	8.33	-1.41	-3.43	-4.29
M	49	-0.69	8.55	-1.25	-3.51	-4.77
M	29	-0.71	8.50	-1.30	-3.54	-4.60
M	53	-0.81	8.50	-1.33	-3.65	-4.70
M	50	-0.84	8.40	-1.41	-3.53	-4.48
M	17	-0.95	8.23	-1.57	-3.61	-4.42
B	09	-0.41	8.46	-1.29	-2.90	-3.82
B	14	-0.32	8.55	-1.21	-2.81	-3.83

F. Interstitial N and substitutional N (N_i–N_O)

During the transformation of LTO to LTON N may be incorporated in the vicinity of already incorporated N atoms. One possibility is a combination of an interstitial N and a substitutional N, which we model by fixing the N_O at its most stable site and sampling different positions for the N_i as shown in Fig. S29. The formation energy decreases with increasing N_i–N_O distance (Fig. S27 and Table S8), which, especially for lower charge states, can be rationalized with N–N bond formation with a bond length close to the N–N single bond in hydrazine. This suggests that anion-anion coupling takes place and stabilizes the relevant configurations, which is also supported by peaks with large N character at the bottom of the VB for $(N_i-N_O)^0$ in Fig. S26a. Unsurprisingly, the length of the N–N bonds increases with increasing charge of the defect complex from 1.2 Å for $(N_i-N_O)^0$ to 1.4 Å for $(N_i-N_O)^{-4}$, which is the most stable charge state for $E_{\mathrm{Fermi}} = E_g^{\mathrm{expt}}$ (Fig. S28a). Interestingly for this charge state, relevant under experimental conditions, defect configurations with N_i lying in the middle layer at larger distances from N_O can be as stable as configurations stabilized by the formation of N–N bonds (Fig. S27d). The formation energy of N_i–N_O decreases with decreasing $\Delta\mu_O$, N_i–N_O defects becoming more stable than single N-related defects in LTO for $\Delta\mu_O < 2$ eV when N_2 is used as reference state for the N chemical potential.

TABLE S8: Formation energy (in eV) for a neutral $(N_i-N_O)^0$, doubly negatively $(N_i-N_O)^{-2}$, singly negatively $(N_i-N_O)^{-1}$), and fourfold negatively $(N_i-N_O)^{-4}$ charged interstitial nitrogen - substitutional nitrogen complex computed for inequivalent N_i sites in the interlayer (I), middle (M), or bulk (B) layer, while N_O is kept fixed at the most stable position (cf. Fig. S29 for site labels).

Layer	Site	$N_i-N_O{}^0$	$N_i-N_O{}^{-2}$	$N_i-N_O{}^{-1}$	$N_i-N_O{}^{-4}$
I	11	4.69	0.75	2.09	-1.97
I	03	1.88	-1.37	0.09	-3.19
I	07	5.02	0.17	2.61	-1.61
M	12	4.27	-0.62	1.45	-2.43
M	06	3.92	-0.37	1.53	-3.13
M	16	5.43	0.25	2.73	-1.58
B	19	5.60	0.50	2.72	-1.33

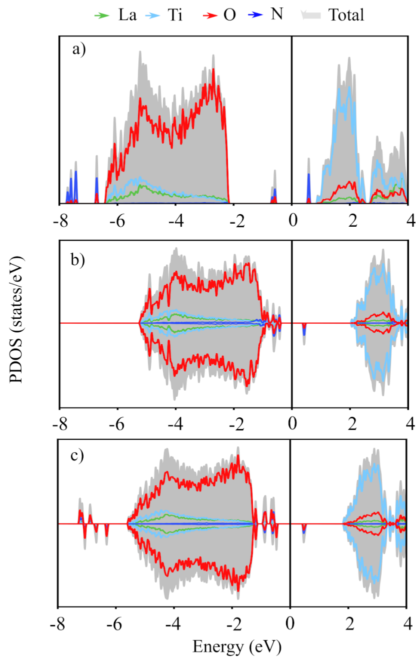

FIG. S26: Total and atom-projected density of states (PDOS) for a) $N_i-N_O{}^0$, b) $N_i-N_O{}^{-1}$, and c) $N_i-N_O{}^{-4}$. For each defect, the origin of the energy scale was set to the respective Fermi energy.

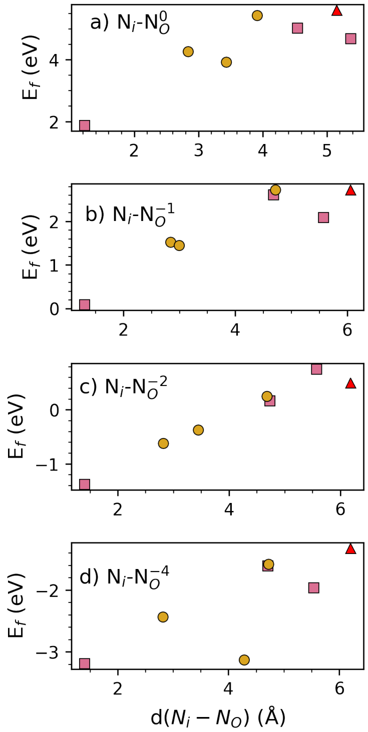

FIG. S27: Formation energy of the different configurations for a) $(N_i-N_O)^0$, b) $(N_i-N_O)^{-1}$, c) $(N_i-N_O)^{-2}$, and d) $(N_i-N_O)^{-4}$ defects in LTO as a function of the distance between N_i and N_O. Pink squares, golden circles, and red triangle correspond to N_i lying in the interlayer, middle, or bulk layer as depicted in Fig. S29 while N_O is kept fixed at the most stable position in the middle layer of the slab.

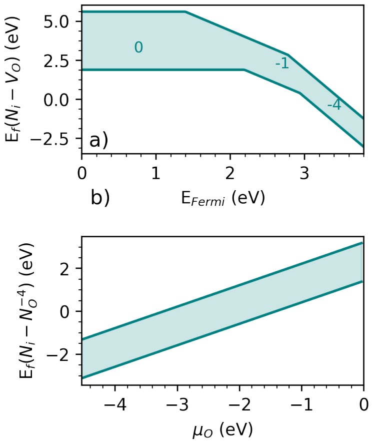

FIG. S28: a) N_i-N_O formation energy ($E_f(\mathrm{N_i}-\mathrm{N_O})$) in different charge states computed as a function of the Fermi energy (E_{Fermi}, up to the experimental band gap E_g^{expt}) referenced to the valence band maximum of LTO and in the O-poor limit ($\Delta\mu_O$=-4.54 eV). Only the most stable charge state is reported and in region of the plot. b) Formation energy for a $(N_i-N_O)^{-4}$ as a function of the oxygen chemical potential and for $E_{\mathrm{Fermi}} = E_g^{\mathrm{expt}}$. In both cases, the colored area indicates the variation of $E_{\mathrm{f}}(\mathrm{N_i}-\mathrm{N_O})$ for different configurations.

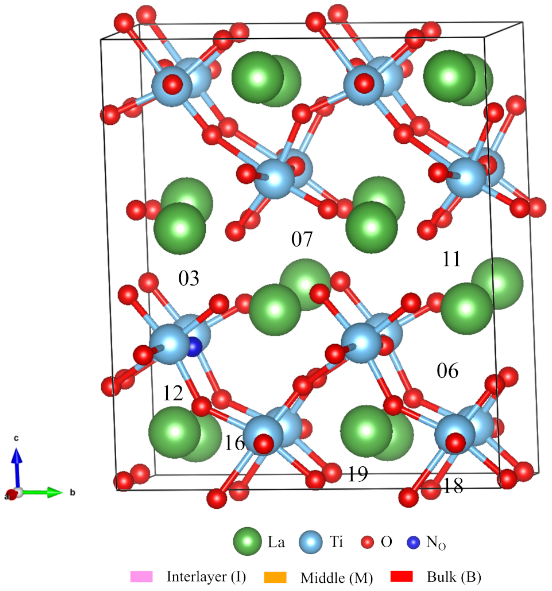

FIG. S29: $1 \times 2 \times 1$ supercell used for modeling $N_i - N_O$ in LTO. Oxygen atoms colored in pink, orange, and red correspond to O lying in the interlayer (I), in the middle (M) or bulk (B) layer. The numbers indicate the different sites for a nitrogen interstitial N_i.

G. Two substitutional N ($N_O - N_O$)

Another possibility to incorporate N in presence of already incorporated N is as two substitutional N. We also fix one N_O in either of three of the most favorable positions (see Table S9 for details) and sample other positions for the second N_O. For the electron deficient $(N_O - N_O)^0$ charge state, configurations in which N−N bonds can be formed are the most stable (Fig. S31a). These are characterized by two occupied N-dominated states in the band gap (Fig. S30a). For the $(N_O - N_O)^{-2}$ charge state most relevant under experimental conditions (Fig. S32a) configurations with larger N−N distances and the second N_O defect lying either in the middle or bulk layer are most favorable (Fig. S31b). For these configurations the N-dominated states lie just above the valence band (Fig. S30b).

Comparing the formation energies of $N_i - N_O$ and $N_O - N_O$, the latter becomes more favorable under $\Delta\mu_O$ smaller than -3 eV. These results suggest that even when N is already substituted in the lattice, further N insertion in LTO can favorably happen, in particular under O-poor conditions, with N entering as interstitial in the interlayer and eventually substituting for O, especially in the middle layer.

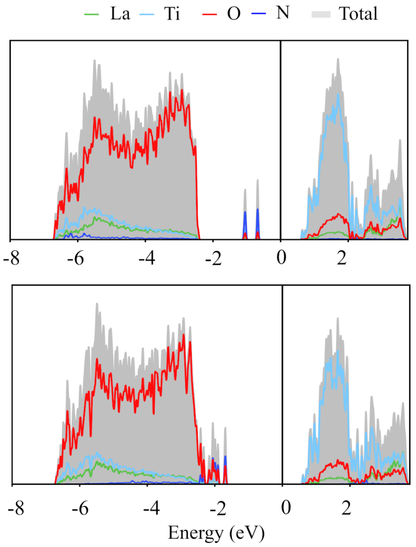

FIG. S30: Total and atom-projected density of states (PDOS) for a) $(N_O - N_O)^0$, and b) $(N_O - N_O)^{-2}$. For each defect, the origin of the energy scale was set at the respective Fermi energy.

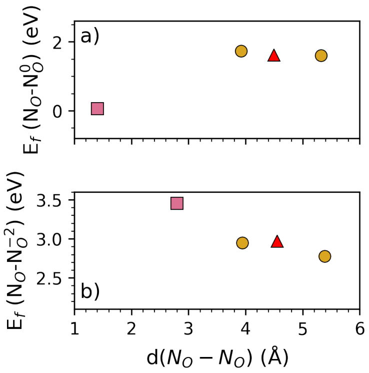

FIG. S31: Formation energy of the different configurations for a) $(N_O - N_O)^0$, and b) $(N_O - N_O)^{-2}$ defects in LTO as a function of the $N_O - N_O$ distance. Pink squares, golden circles, and red triangle correspond to one N_O lying in the interlayer, middle, or bulk layer as depicted in Fig. S33 while the second N_O is kept fixed at the most stable position in the middle layer of the slab.

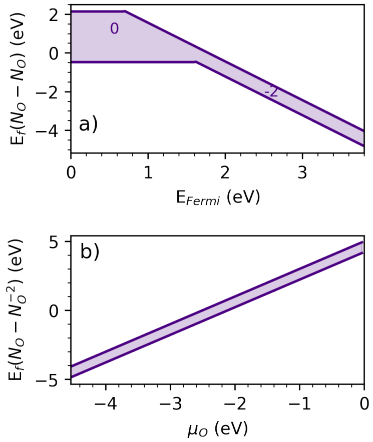

FIG. S32: a) $N_O - N_O$ formation energy ($E_f(N_O - N_O)$) in different charge states computed as a function of the Fermi energy (E_{Fermi}, up to the experimental band gap E_g^{expt}) referenced to the valence band maximum of LTO and in the O-poor limit ($\Delta\mu_O$=-4.54 eV). Only the most stable charge state is reported and in region of the plot. b) Formation energy for a $(N_O - N_O)^{-2}$ as a function of the oxygen chemical potential and for $E_{\mathrm{Fermi}} = E_g^{\mathrm{expt}}$. In both cases, the colored area indicates the variation of $E_{\mathrm{f}}(N_O - N_O)$ for different configurations.

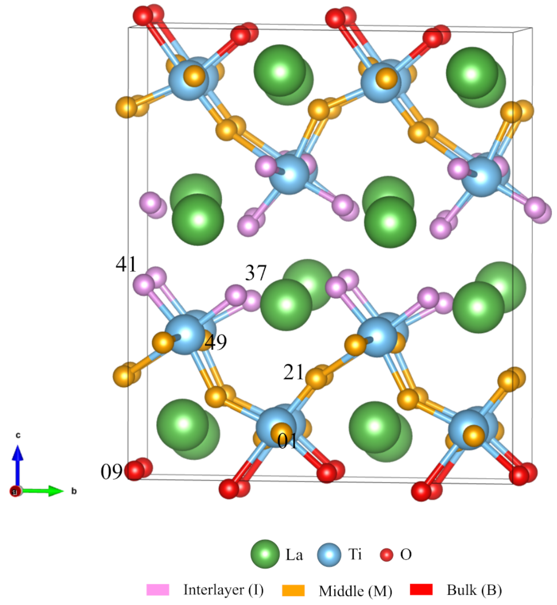

FIG. S33: $1 \times 2 \times 1$ supercell used for modeling N_O-N_O in LTO. Oxygen atoms colored in pink, orange, and red correspond to O lying in the interlayer, in the middle or bulk layer. The numbers indicate the different sites for a substitutional nitrogen N_O while the second N_O is fixed on either of three of the most favorable position in the interlayer (see Table S9 for details).

TABLE S9: Formation energy (in eV) for a neutral $(N_O-N_O)^0$, doubly negatively $(N_O-N_O)^{-2}$, and singly negatively $(N_O-N_O)^{-1}$ charged complex made by two substitutional nitrogen defects in different configurations created by fixing one N_O in three different positions close to the interface (sites 37, 41, and 49 in Fig. S33 and indicated by the number preceding the N in the site labels reported in this Table), while the second N_O is positioned in one of the other possible sites labeled in Fig. S33 and lying in the interlayer (I), middle (M), or bulk (B) layer.

Layer	Site	N_O-$N_O{}^0$	N_O-$N_O{}^{-2}$	N_O-$N_O{}^{-1}$
I	49N-37	0.06	3.45	2.32
I	49N-41	-0.15	3.55	2.18
M	41N-01	2.07	3.04	2.35
M	49N-21	1.73	2.95	1.95
M	37N-01	2.00	2.98	2.21
M	41N-21	2.14	3.18	2.42
M	37N-21	-0.48	3.11	2.21
M	41N-49	-0.15	3.55	2.18
M	37N-49	0.06	3.45	2.32
M	49N-01	1.61	2.77	1.83
B	37N-09	2.08	3.17	2.36
B	49N-09	1.62	2.97	2.00
B	41N-09	2.01	3.20	2.35

H. N diffusion

Table S10 reports the largest migration barriers for the paths shown in Fig. S34.

TABLE S10: Largest migration barrier (in eV) along the paths of Fig. S34 for N migration via a neutral $(V_O-N_O)^0$, singly positively $(V_O-N_O)^{+1}$, doubly positively $(V_O-N_O)^{+2}$ or singly negatively $(V_O-N_O)^{-1}$ charged oxygen vacancy-nitrogen substitutional defect pair.

Path	$(V_O-N_O)^0$	$(V_O-N_O)^{+1}$	$(V_O-N_O)^{+2}$	$(V_O-N_O)^{-1}$
Pink	0.52	0.81	0.01	0.89
Orange	1.28	1.96	0.50	1.99
Purple	1.67	1.12	0.77	1.32
Green	1.67	1.12	0.77	1.32
Blue	1.86	1.96	1.32	-
Red	1.09	0.96	0.78	-

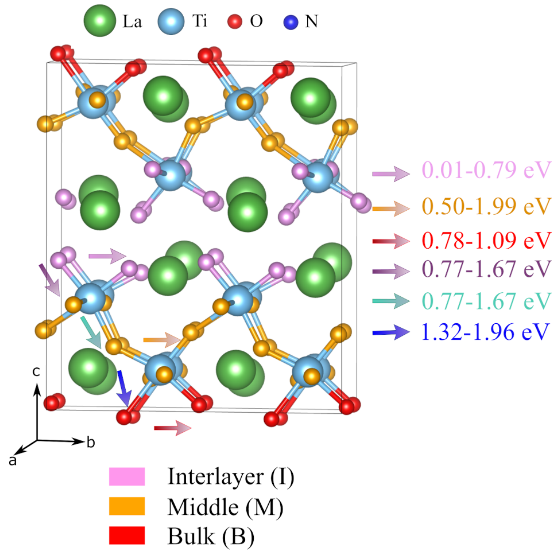

FIG. S34: Schematic representation of the different paths considered for N diffusion in LTO represented by the colored arrows. The numerical values on the right correspond to the range of possible activation energy barriers for diffusion along the corresponding path.

S5. DEFECT CHEMISTRY OF LTON

A. Oxygen (V_O) and nitrogen (V_N) vacancies

Doubly (${V_O}^{+2}$), singly (${V_O}^{+1}$) positively charged and neutral oxygen (${V_O}^{0}$) vacancies can be formed in LTON in the two symmetry inequivalent configurations shown in Fig. S35, one breaking a Ti−O−Ti bond within the *ac* plane containing the N atoms (in-plane, IP) and one breaking a Ti−O−Ti bond perpendicular to this plane (out-of-plane, OP). For a neutral defect the two extra electrons are localized on the vacancy site and occupy a state with Ti-character at the bottom of the conduction band (Fig. S36a). In this case the two configurations show very similar formation energies (Fig. S37), while larger differences are observed for charged defects with the IP configuration being lower by 0.23 and 0.47 eV for ${V_O}^{+1}$ and ${V_O}^{+2}$ respectively (Table S11). This can be rationalized by the different electrostatic interactions ($N^{-3}-Ti^{+4}-{V_O}^{IP}-Ti^{+4}-N^{-3}$ or $O^{-2}-Ti^{+4}-{V_O}^{OP}-Ti^{+4}-O^{-2}$) taking place in the two cases.

Interestingly, when computed for the same synthesis conditions, the formation energy of an oxygen vacancy in LTON is about 1 eV higher compared to LTO. This suggests that in coexistence of the two materials, during the transformation of LTO to LTON, oxygen vacancies will preferentially be formed in the oxide where they can be annihilated by N, favoring further conversion to LTON.

Furthermore, in the N-rich environment of the thermal ammonolysis, formation of oxygen vacancies is much more favorable than the formation of nitrogen vacancies (V_N), compare Figs. S37 and S39 as well as Tables S11 and S12) by as much as 1-2 eV for $E_{\mathrm{Fermi}}= E_g^{\mathrm{expt}}$ and $\Delta\mu_O$=-4.54 eV. Not only is this important for the transformation of LTO to LTON, but the formation of V_N would also result in the appearance of more pronounced in-gap states compared to V_O (Figs. S38 and S36) which could be detrimental for the photocatalytic performance of LTON.

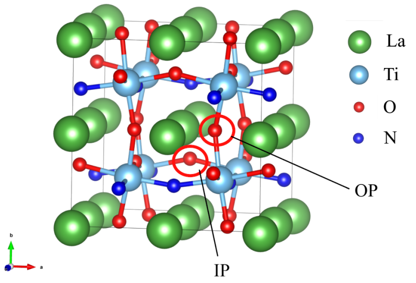

FIG. S35: Inequivalent oxygen-vacancy sites in LTON, one breaking a Ti−O−Ti bond within the *ac* plane containing the N atoms (IP) and one breaking a Ti−O−Ti bond perpendicular to this plane (OP).

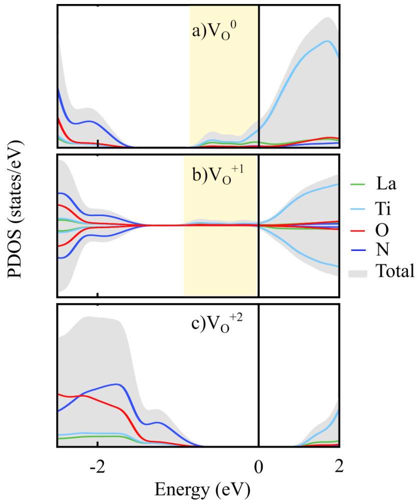

FIG. S36: Total and atom-projected density of states (PDOS) for a) ${V_O}^0$, b) ${V_O}^{+1}$, and c) ${V_O}^{+2}$ in LTON. For each defect, the origin of the energy scale was set at the respective Fermi energy system. The area highlighted in yellow indicates the defect state.

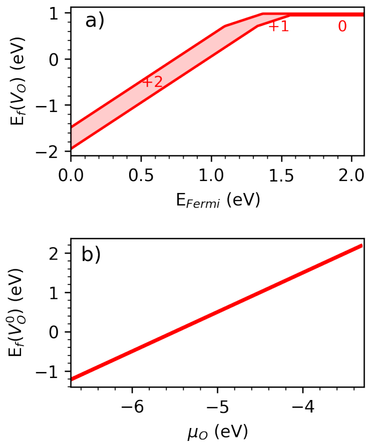

FIG. S37: a) Oxygen vacancy formation energy ($E_f(V_O)$) in different charge states computed as a function of the Fermi energy (E_{Fermi}, up to the experimental band gap E_g^{expt}) referenced to the valence band maximum of LTON and for $\Delta\mu_O$=-4.54 eV. Only the most stable charge state is reported in each region of the plot. b) Formation energy for a neutral oxygen vacancy as a function of the oxygen chemical potential and for $E_{\mathrm{Fermi}} = E_g^{\mathrm{expt}}$. The colored area indicates the variation of $E_f(V_O)$ for different configurations.

TABLE S11: Formation energy (in eV) for a neutral ($V_O{}^0$), singly positively ($V_O{}^{+1}$), and doubly positively charged ($V_O{}^{+2}$) oxygen vacancy computed for the IP and OP positions in the LTON cell (see. Fig. S35 for site labels).

Conf.	$V_O{}^0$	$V_O{}^{+1}$	$V_O{}^{+2}$
OP	0.98	1.71	2.72
IP	0.94	1.48	2.25

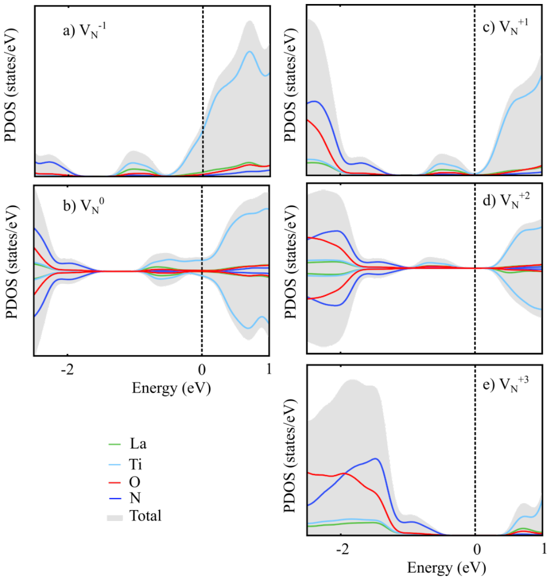

FIG. S38: Total and atom-projected density of states (PDOS) for a) $V_N{}^{-1}$, b) $V_N{}^0$, c) $V_N{}^{+1}$, d) $V_N{}^{+2}$, and e) $V_N{}^{+3}$ in LTON. For each defect, the origin of the energy scale was set at the respective Fermi energy.

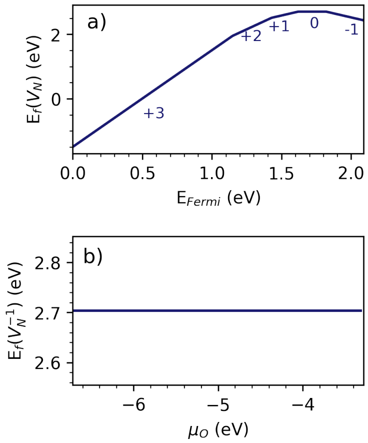

FIG. S39: a) Nitrogen vacancy formation energy ($E_f(V_N)$) in different charge states computed as a function of the Fermi energy (E_{Fermi}, up to the experimental band gap E_g^{expt}) referenced to the valence band maximum of LTON and for $\Delta\mu_O$=-4.54 eV. Only the most stable charge state is reported in each region of the plot. b) Formation energy for a $V_N{}^{-1}$ as a function of the oxygen chemical potential and for $E_{Fermi} = E_g^{expt}$. The colored area indicates the variation of $E_f(V_N)$ for different configurations.

TABLE S12: Formation energy (in eV) for a singly negatively charged ($V_N{}^{-1}$), neutral ($V_N{}^0$) as well as singly ($V_N{}^{+1}$), doubly ($V_N{}^{+2}$), and triply ($V_N{}^{+3}$) positively charged nitrogen vacancy in LTON.

$V_N{}^{-1}$	$V_N{}^0$	$V_N{}^{+1}$	$V_N{}^{+2}$	$V_N{}^{+3}$
2.42	2.70	3.18	3.86	3.48

B. Substitutional nitrogen (N_O) and oxygen (O_N)

Substitutional nitrogen (N_O) are among the most stable defects in LTON under the considered N-rich conditions (Fig. S41 and Table S13). The formation of N_O does not lead to in-gap states which could be detrimental to the photocatalytic performance of LTON (Fig. S40). N prefers to be inserted in IP positions in Fig. S35 supporting that, when nitrogen is already present, further N introduction is favored in a *cis* arrangement due to the bonding optimization between N/O and the Ti atoms compared to the *trans* order resulting from substitution at OP sites.

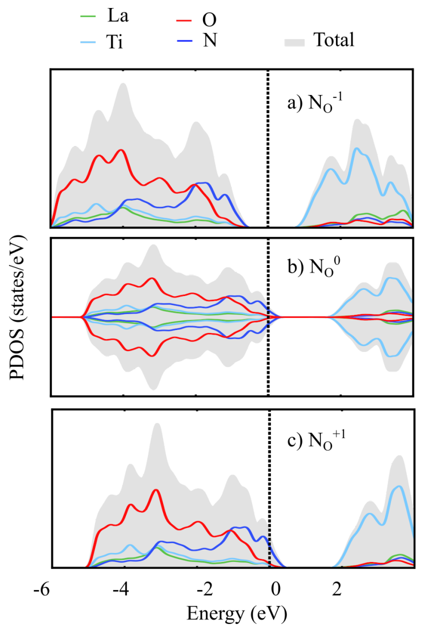

FIG. S40: Total and atom-projected density of states (PDOS) for a) $N_O{}^{-1}$, b) $N_O{}^{0}$, and c) $N_O{}^{+1}$ in LTON. For each defect, the origin of the energy scale was set at the respective Fermi energy.

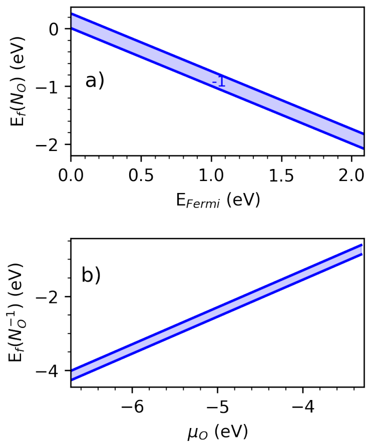

FIG. S41: a) Substitutional nitrogen formation energy ($E_f(N_O)$) in different charge states computed as a function of the Fermi energy ($E_{\rm Fermi}$, up to the experimental band gap $E_g^{\rm expt}$) referenced to the valence band maximum of LTON and for $\Delta\mu_O$=-4.54 eV. Only the most stable charge state is reported in each region of the plot. b) Formation energy for a $N_O{}^{-1}$ as a function of the oxygen chemical potential and for $E_{\rm Fermi} = E_g^{\rm expt}$. The colored area indicates the variation of $E_{\rm f}(N_O)$ for different configurations.

TABLE S13: Formation energy (in eV) for a neutral ($N_O{}^{0}$), singly positively ($N_O{}^{+1}$), and singly negatively charged ($N_O{}^{-1}$) substitutional nitrogen computed for the IP and OP positions in LTON (see Fig. S35 for site labels).

Conf.	$N_O{}^{0}$	$N_O{}^{+1}$	$N_O{}^{-1}$
IP	0.19	2.44	-2.09
OP	0.26	2.44	-1.84

Substitutional O at a nitrogen site prefers the -1 charge state and is possible only under O-richer conditions, in particular for $\Delta\mu_{O} > -4.1$ eV, where it becomes the most stable O-related defect in LTON (Fig. S43 and Table S14). These defects do not lead to in-gap states and do not negatively affect the photocatalytic performance of LTON (Fig. S42).

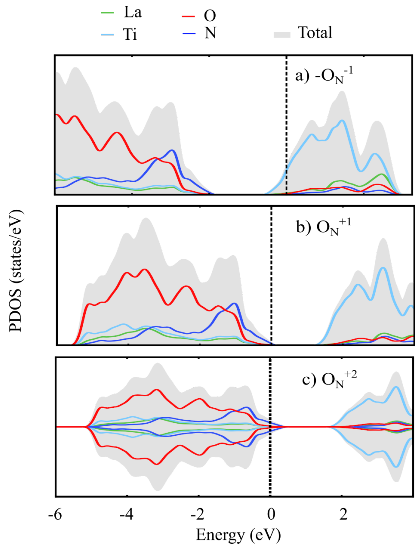

FIG. S42: Total and atom-projected density of states (PDOS) for a) $O_N{}^{-1}$, b) $O_N{}^{+1}$, and c) $O_N{}^{+2}$ in LTON. For each defect, the origin of the energy scale was set at the respective Fermi energy.

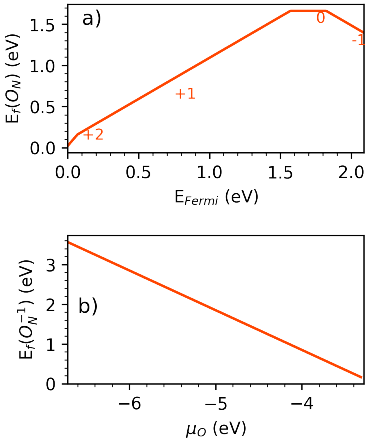

FIG. S43: a) Substitutional oxygen formation energy ($E_f(O_N)$) in different charge states computed as a function of the Fermi energy (E_{Fermi}, up to the experimental band gap E_g^{expt}) referenced to the valence band maximum of LTON and for $\Delta\mu_O$=-4.54 eV. Only the most stable charge state is reported in each region of the plot. b) Formation energy for a $O_N{}^{-1}$ as a function of the oxygen chemical potential and for $E_{\mathrm{Fermi}} = E_g^{\mathrm{expt}}$. The colored area indicates the variation of $E_{\mathrm{f}}(O_N)$ for different configurations.

TABLE S14: Formation energy (in eV) for a singly negatively charged ($O_N{}^{-1}$), neutral ($O_N{}^{0}$) as well as singly ($O_N{}^{+1}$), doubly ($O_N{}^{+2}$), and triply ($O_N{}^{+3}$) positively charged substitutional oxygen in LTON.

$O_N{}^{-1}$	$O_N{}^{0}$	$O_N{}^{+1}$	$O_N{}^{+2}$	$O_N{}^{+3}$
1.39	1.66	2.19	4.22	3.87

These results on substitutional atoms again underline the importance of the synthesis environment, a N-rich environment from which O is continuously removed being pivotal to favor LTON formation.

C. Interstitial nitrogen (N_i) and oxygen (O_i)

For nitrogen and oxygen interstitials we sampled the positions shown in Fig. S44. The interstitials generally prefer to be located at distances of more than 2.5 Å from the nearest anion (Fig. S46 and in the experimentally most relevant -3 charge state (Fig. S47a and Table S15) lead to defect states in the band gap (Fig. S45c).

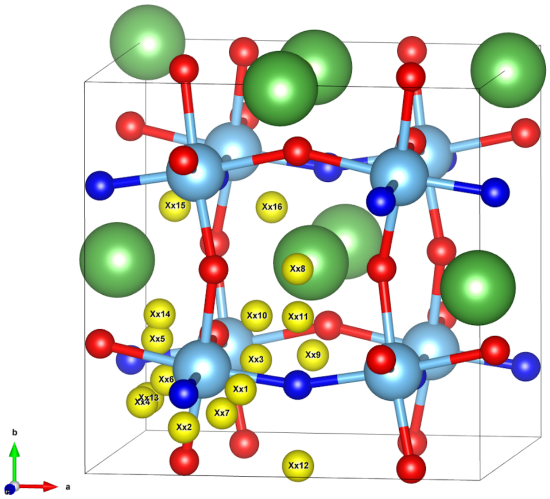

FIG. S44: Initial position tested for the calculation of N and O interstitial defects in LTON. Red, blue, green, and light blue spheres correspond to O, N, La, and Ti atoms, while the yellow numbered spheres indicate the different sampled N_i and O_i positions.

TABLE S15: Formation energy (in eV) for a neutral (N_i^0), triply (N_i^{-3}) and singly (N_i^{-1}) negatively charged nitrogen interstitial in LTON. Labels for the different configurations corresponds to the initial interstitial position of Fig. S44.

Conf.	N_i^0	N_i^{-3}	N_i^{-1}
1	3.75	0.92	1.55
2	2.59	-1.33	1.19
3	2.91	0.30	1.45
4	4.58	1.16	2.43
5	4.72	0.96	2.64
6	4.72	1.08	2.73
7	3.75	-0.36	1.51
8	5.09	0.67	2.93
9	3.67	0.26	3.49
10	4.72	1.53	2.68
11	2.59	-0.17	1.19
12	2.59	-0.17	1.20
13	5.09	1.56	2.82
14	4.72	1.53	2.67
15	2.91	-0.17	1.20
16	3.75	0.24	1.55

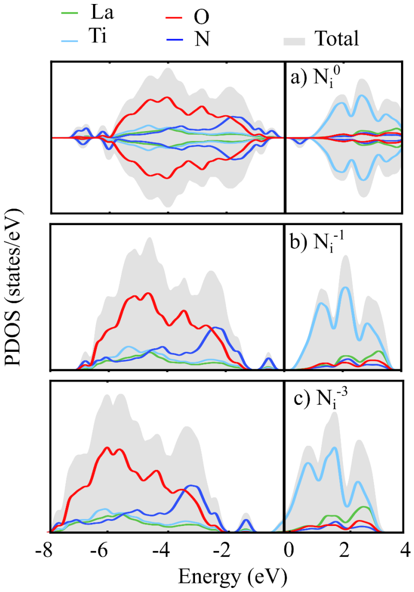

FIG. S45: Total and atom-projected density of states (PDOS) for a) ${N_i}^0$, b) ${N_i}^{-1}$, and c) ${N_i}^{-3}$. For each defect the origin of the energy scale was set at the respective Fermi energy.

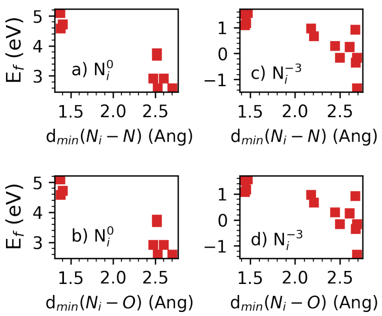

FIG. S46: Formation energy of the different configurations for one ${N_i}^0$/${N_i}^{-3}$ as a function of the distance between N_i and the closest a)/b) N or c)/d) O atom in LTON.

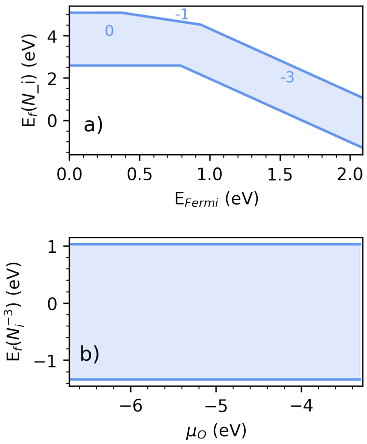

FIG. S47: a) Nitrogen interstitial formation energy ($E_f(\mathrm{N_i})$) in different charge states computed as a function of the Fermi energy (E_{Fermi}, up to the experimental band gap E_g^{expt}) referenced to the valence band maximum of LTON and in the O-poor limit ($\Delta\mu_{\mathrm{O}}$=-4.54 eV). Only the most stable charge state is reported in each region of the plot. b) Formation energy for a ${N_i}^{-3}$ as a function of the oxygen chemical potential and for $E_{\mathrm{Fermi}} = E_g^{\mathrm{expt}}$. In both cases, the colored area indicates the variation of $E_{\mathrm{f}}(\mathrm{N_i})$ for different configurations.

Neutral oxygen interstitials (Fig. S49a) prefer to form close to a nitrogen, forming O−N bonds. This is not the case in the doubly negatively charged oxygen interstitial (Fig. S49b), where configurations with large O−N distances are more stable. It is visible from Figs. S49c and d, though, that in the doubly charged case O−O bonds are preferred, which is not the case for the neutral interstitial. Oxygen intersititials do, in the experimentally relevant -2 charge state (Fig. S50a and Table S16) not induce any gap states (Fig. S48).

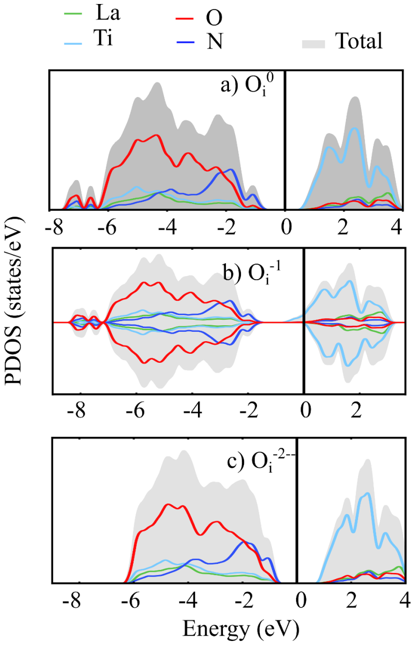

FIG. S48: Total and atom-projected density of states (PDOS) for one a) $O_i{}^0$, b) $O_i{}^{-1}$, and c) $O_i{}^{-3}$. For each defect, the origin of the energy scale was set at the respective Fermi energy.

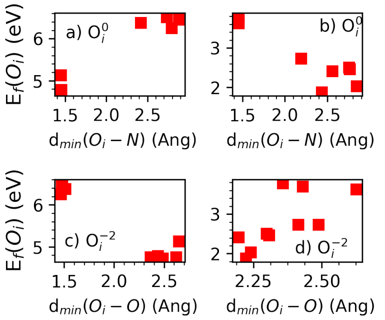

FIG. S49: Formation energy of the different configurations for one $O_i{}^0/O_i{}^{-2}$ as a function of the distance between O_i and the closest a)/b) N or c)/d) O atom in LTON.

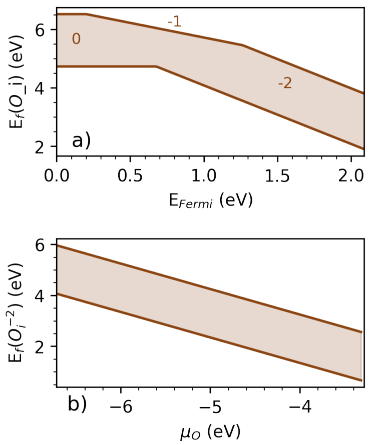

FIG. S50: a) Oxygen interstitial formation energy ($E_f(\mathrm{O_i})$) in different charge states computed as a function of the Fermi energy (E_{Fermi}, up to the experimental band gap E_g^{expt}) referenced to the valence band maximum of LTON and in the O-poor limit ($\Delta\mu_{\mathrm{O}}$=-4.54 eV). Only the most stable charge state is reported in each region of the plot. b) Formation energy for a $O_i{}^{-2}$ as a function of the oxygen chemical potential and for $E_{\mathrm{Fermi}} = E_g^{\mathrm{expt}}$. In both cases, the colored area indicates the variation of $E_{\mathrm{f}}(\mathrm{N_i})$ for different configurations.

TABLE S16: Formation energy (in eV) for a neutral (${O_i}^0$), doubly (${O_i}^{-2}$) and singly (${O_i}^{-1}$) negatively charged oxygen interstitial in LTON. Labels for the different configurations corresponds to the initial interstitial position of Fig. S44.

Conf.	${O_i}^0$	${O_i}^{-2}$	${O_i}^{-1}$
1	5.13	2.73	4.54
2	4.73	3.62	4.15
3	4.76	3.78	4.24
4	6.26	2.04	4.29
5	6.46	2.47	4.62
6	6.45	2.47	4.62
7	5.13	1.88	4.47
8	6.52	2.04	4.41
9	5.13	2.50	4.28
10	6.38	1.88	4.28
11	4.78	3.70	4.18
12	4.75	3.62	4.13
13	6.52	2.41	4.29
14	6.45	2.47	4.62
15	4.76	3.78	4.28
16	5.13	2.73	4.47

D. Interstitial nitrogen and oxygen vacancy ($\mathbf{N}_i-\mathbf{V_O}$)

In addition to isolated interstitial N and O, we also considered complexes with oxygen vacancies (V_O). The N_i-V_O complex prefers a -3 charge state under experimentally relevant conditions (Fig. S51a and Table S17) and becomes more stable than an isolated N_i only under very O-poor conditions (Fig. S51b).

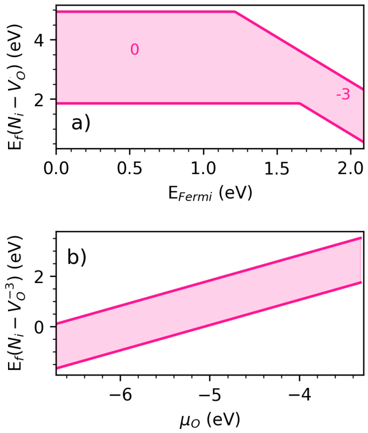

FIG. S51: a) Oxygen vacancy-nitrogen interstitial complex formation energy ($E_f(\mathrm{N_i}-\mathrm{V_O})$) in different charge states computed as a function of the Fermi energy (E_{Fermi}, up to the experimental band gap E_g^{expt}) referenced to the valence band maximum of LTON and in the O-poor limit ($\Delta\mu_{\mathrm{O}}$=-4.54 eV). Only the most stable charge state is reported in each region of the plot. b) Formation energy for a $(\mathrm{N}_i-\mathrm{V_O})^{-3}$ complex as a function of the oxygen chemical potential and for $E_{\mathrm{Fermi}} = E_g^{\mathrm{expt}}$. In both cases, the colored area indicates the variation of $E_{\mathrm{f}}(\mathrm{N_i}-\mathrm{V_O})$ for different configurations.

TABLE S17: Formation energy (in eV) for a neutral $(N_i{-}V_O)^0$, singly $(N_i{-}V_O)^{-1}$, doubly $(N_i{-}V_O)^{-2}$, and triply $(N_i{-}V_O)^{-3}$ negatively charged nitrogen interstitial-oxygen vacancy defect pair. The 32 tested configurations are obtained combining an IP or OP V_O with the 16 N_i positions of Fig. S44.

Conf.	$(N_i{-}V_O)^0$	$(N_i{-}V_O)^{-1}$	$(N_i{-}V_O)^{-2}$	$(N_i{-}V_O)^{-3}$
1	2.41	2.56	0.93	1.39
2	2.23	1.65	1.01	0.62
3	2.23	1.65	1.23	0.52
4	0.48	-1.68	-2.60	-2.92
5	3.55	3.68	2.79	1.97
6	3.70	3.68	3.02	1.97
7	4.94	4.51	3.40	1.39
8	0.17	-2.13	-2.61	-3.35
9	4.62	4.38	3.61	1.50
10	0.17	-2.09	-2.86	-3.35
11	2.23	1.65	0.98	0.52
12	2.23	1.65	0.98	0.52
13	0.26	3.23	3.30	2.29
14	0.17	3.68	3.09	1.97
15	2.05	1.72	1.03	0.73
16	2.51	1.72	1.03	0.73
17	1.85	-1.85	1.04	0.76
18	1.82	-1.84	1.01	0.90
19	2.91	2.33	1.76	1.15
20	0.26	-1.84	-2.58	-3.08
21	0.26	-1.85	-2.41	-3.10
22	0.26	-1.85	-2.41	-3.10
23	1.85	2.33	1.62	1.09
24	3.60	-2.14	-2.86	1.21
25	4.66	-1.85	3.64	0.76
26	0.26	-1.85	-2.79	-3.10
27	2.93	2.21	1.41	1.14
28	2.81	2.20	1.57	1.14
29	0.26	-1.85	-2.68	-3.10
30	0.26	-1.85	-2.68	-3.10
31	2.78	2.08	1.05	0.88
32	2.83	2.45	1.88	1.64

E. Interstitial oxygen and oxygen vacancy (O_i–V_O)

The O_i–V_O Frenkel pair leads to doping into the conduction band (Fig. S52) in the experimentally relevant -2 charge state (Fig. S54a and Table S18). Generally configurations with larger O_i–N or O_i–O separation are preferred, except for $(O_i–V_O)^{-1}$, where intermediate distances are more favorable (Fig. S53). Compared to the isolated O_i, the O_i–V_O is favorable under O-poor conditions for $\Delta\mu_O$=-4.5 eV.

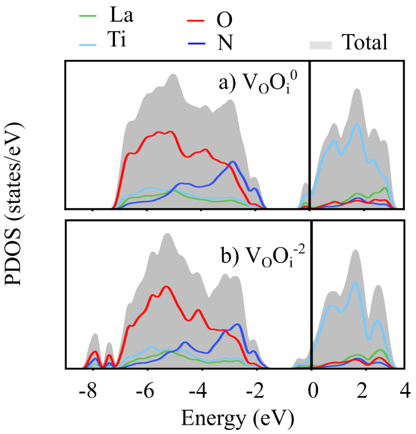

FIG. S52: Total and atom-projected density of states (PDOS) for a) $(V_O–O_i)^0$, and b) $(V_O–O_i)^{-2}$. For each defect, the origin of the energy scale was set at the respective Fermi energy.

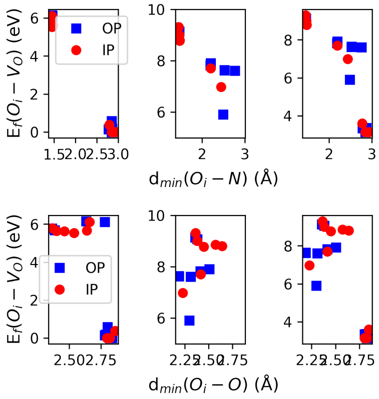

FIG. S53: Formation energy of the different configurations for one O_i–V_O as a function of the distance between O_i and the closest N (top) or O atom (bottom) for a $(O_i–V_O)^0$ (left), $(O_i–V_O)^{-1}$ (center), and $(O_i–V_O)^{-2}$ (right) in LTON. Red circles and blue squares are for configurations with an IP and OP V_O, respectively.

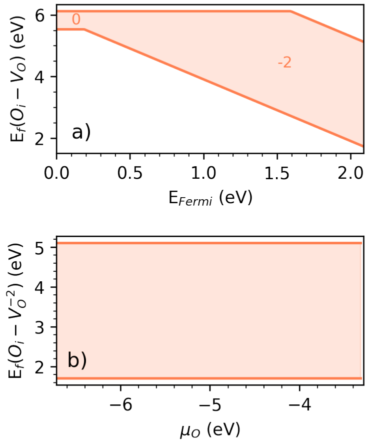

FIG. S54: a) O_i–V_O formation energy ($E_f(O_i - V_O)$) in different charge states computed as a function of the Fermi energy (E_{Fermi}, up to the experimental band gap E_g^{expt}) referenced to the valence band maximum of LTON and in the O-poor limit ($\Delta\mu_O$=-4.54 eV). Only the most stable charge state is reported in each region of the plot. b) Formation energy for a $(O_i–V_O)^{-2}$ as a function of the oxygen chemical potential and for $E_{Fermi} = E_g^{expt}$. In both cases, the colored area indicates the variation of $E_f(O_i - V_O)$ for the different configurations.

TABLE S18: Formation energy (in eV) for a neutral $(O_i{-}V_O)^0$, doubly $(O_i{-}V_O)^{-2}$, and singly $(O_i{-}V_O)^{-1}$ negatively charged oxygen vacancy-oxygen interstitial defect pair. The 32 tested configurations are obtained combining an IP or OP V_O with the 16 O_i positions of Fig. S44.

Conf.	$(O_i{-}V_O)^0$	$(O_i{-}V_O)^{-2}$	$(O_i{-}V_O)^{-1}$
1	6.12	7.91	7.91
2	0.14	3.34	3.34
3	5.77	9.14	9.14
4	0.18	3.35	3.35
5	0.00	7.61	7.61
6	0.18	7.61	7.61
7	6.12	5.91	5.91
8	-0.01	3.14	3.14
9	0.57	7.64	7.64
10	-0.01	3.14	3.14
11	-0.01	3.14	3.14
12	0.14	3.34	3.34
13	0.18	7.62	7.62
14	0.00	3.17	3.17
15	5.69	9.07	9.07
16	6.16	7.83	7.83
17	0.37	3.60	3.60
18	5.53	8.86	8.86
19	5.64	9.00	9.00
20	-0.00	3.14	3.14
21	-0.01	3.14	3.14
22	-0.01	3.14	3.14
23	5.64	6.98	6.98
24	-0.01	3.14	3.14
25	-0.01	3.14	3.14
26	-0.01	3.14	3.14
27	5.63	8.78	8.78
28	5.65	8.81	8.81
29	-0.01	3.14	3.14
31	5.78	9.31	9.31
30	-0.01	3.14	3.14
32	6.11	7.70	7.70

F. La_{Ti} cation antisites and complexes

La and Ti-related defects are not directly involved in the transformation of LTO to LTON, but they could form during the process, affecting it or influencing the properties of the resulting oxynitride. We considered both antisite defects (La_{Ti} and Ti_{La}) and neutral defect pairs ($La_{Ti}-O_N$ and $Ti_{La}-N_O$).

For La_{Ti}, the most relevant charge state under experimental conditions is ${La_{Ti}}^{-2}$, for which we observe filling of the states at the bottom of the CB (Fig. S55d). ${La_{Ti}}^{-2}$ could be easily formed under Ti-poor and La-rich conditions independently of $\mu_{O/N}$ (Fig. S56b and Table S19). In Ti-poor, La-rich and O-rich environments, the formation of $La_{Ti}-O_N$ could also take place (Fig. S58), defect pair configurations in which La_{Ti} and O_N are close to each other being most favored (Fig. S57 and Table S20).

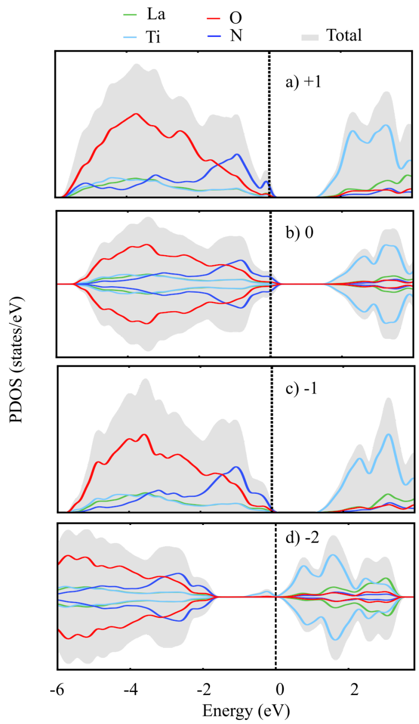

FIG. S55: Total and atom-projected density of states (PDOS) for a) ${La_{Ti}}^{+1}$, b) ${La_{Ti}}^{0}$, c) ${La_{Ti}}^{-1}$, and d) ${La_{Ti}}^{-2}$ in LTON. For each defect, the origin of the energy scale was set at the respective Fermi energy.

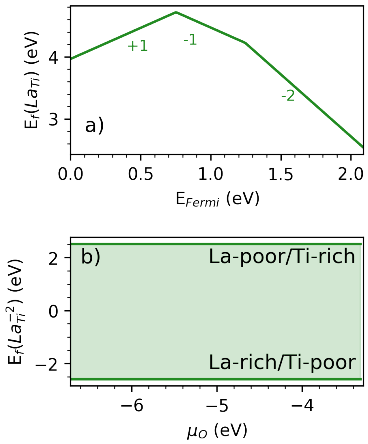

FIG. S56: a) ${La_{Ti}}^{+1}$ antisite formation energy ($E_f(La_{Ti})$) in different charge states computed as a function of the Fermi energy (E_{Fermi}, up to the experimental band gap E_g^{expt}) referenced to the valence band maximum of LTON and for $\Delta\mu_O$=-4.54 eV. Only the most stable charge state is reported in each region of the plot. b) Formation energy for a ${La_{Ti}}^{-2}$ as a function of the oxygen chemical potential and for $E_{Fermi} = E_g^{expt}$. The colored area indicates the range of variation of the formation energy from La-rich/Ti-poor to La-poor/Ti-rich conditions.

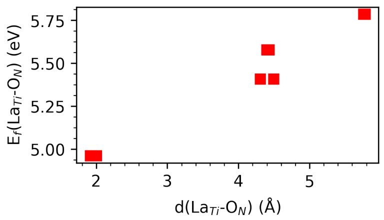

FIG. S57: Formation energy of the different configurations for one $(La_{Ti}-O_N)^0$ defect pair in LTON as a function of the distance between La_{Ti} and O_N.

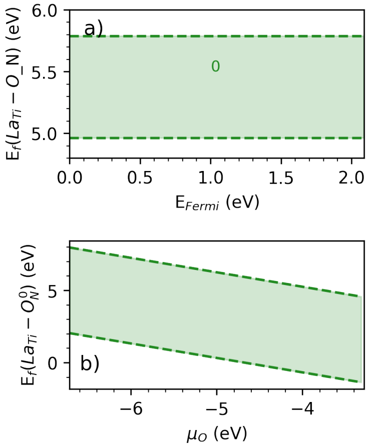

FIG. S58: a) $La_{Ti}-O_N$ formation energy ($E_f(La_{Ti}-O_N)$) in the neutral charge state computed as a function of the Fermi energy (E_{Fermi}, up to the experimental band gap E_g^{expt}) referenced to the valence band maximum of LTON and for $\Delta\mu_O$=-4.54 eV. b) Formation energy for a $(La_{Ti}-O_N)^0$ as a function of the oxygen chemical potential and for $E_{Fermi}=E_g^{expt}$. The colored area indicates the variation of $E_f(La_{Ti}-O_N)$ for different configurations.

TABLE S19: Formation energy (in eV) for a singly negatively charged (${La_{Ti}}^{-1}$), neutral (${La_{Ti}}^0$), singly (${La_{Ti}}^{+1}$), and doubly (${La_{Ti}}^{+2}$) positively charged La substitutional at a Ti site in LTON.

${La_{Ti}}^{-1}$	${La_{Ti}}^{0}$	${La_{Ti}}^{+1}$	${La_{Ti}}^{+2}$
3.37	5.69	6.06	2.52

TABLE S20: Formation energy (in eV) for a neutral $(La_{Ti}-O_N)^0$ and distance between the two defects ($d(La_{Ti}-O_N)$)

Conf.	$d(La_{Ti}-O_N)$	${La_{Ti}-O_N}^0$
1	2.00	4.96
2	4.31	5.41
3	4.39	5.58
4	5.76	5.79
5	4.43	5.58
6	5.78	5.79
7	1.92	4.96
8	4.49	5.41

G. Ti_{La} cation antisites and complexes

Under Ti-rich and La-poor conditions, the formation of Ti_{La} is also possible (Fig. S60b and Table S21). ${Ti_{La}}^0$ is the most relevant charge state under experimental conditions (Fig. S60a) and is associated with the appearance of a filled Ti-3d state at the top of the VB (Fig. S59a). Under O-poor conditions ($\mu_O < -5.7$ eV, Fig. S62 and Table S21), which are relevant for the transformation of LTO to LTON, the $Ti_{La}-N_O$ defect pair becomes favorable, especially for configurations in which the two defects are close to each other and N_O is in IP position (Fig. S61), similarly to what was already observed for a single N_O.

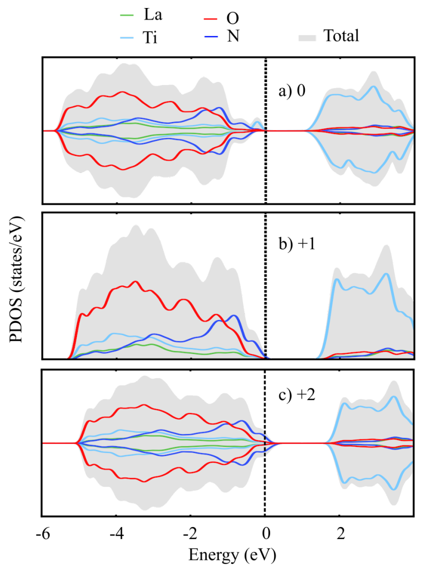

FIG. S59: Total and atom-projected density of states (PDOS) for one a) ${Ti_{La}}^0$, b) ${Ti_{La}}^{+1}$, and c) ${Ti_{La}}^{+2}$ in LTON. For each defect, the origin of the energy scale was set at the respective Fermi energy.

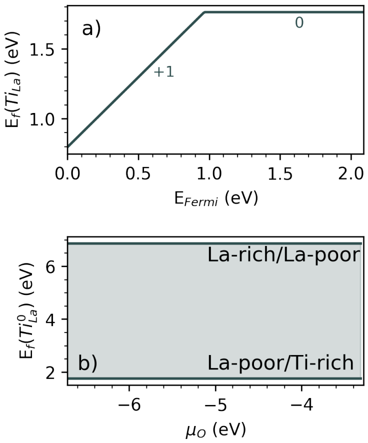

FIG. S60: a) Ti_{La} formation energy ($E_f(Ti_{La})$) in different charge states computed as a function of the Fermi energy (E_{Fermi}, up to the experimental band gap E_g^{expt}) referenced to the valence band maximum of LTON and for $\Delta\mu_O$=-4.54 eV. Only the most stable charge state is reported in each region of the plot. b) Formation energy for a ${Ti_{La}}^0$ as a function of the oxygen chemical potential and for $E_{Fermi}= E_g^{expt}$. The colored area indicates the variation of the formation energy from La-poor/Ti-rich to La-rich/Ti-poor conditions.

TABLE S21: Formation energy (in eV) for a neutral (${Ti_{La}}^0$), singly (${Ti_{La}}^{+1}$), and doubly (${Ti_{La}}^{+2}$) Ti substitutional at a La site in LTON.

${Ti_{La}}^0$	${Ti_{La}}^{+1}$	${Ti_{La}}^{+2}$
1.76	2.90	5.11

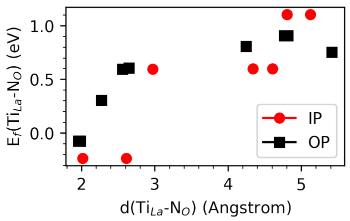

FIG. S61: Formation energy of the different configurations for a $(Ti_{La}-N_O)^0$ defect pair in LTON as a function of the distance between Ti_{La} and N_O. Red circles and black squares are for configurations with and IP and OP N_O, respectively.

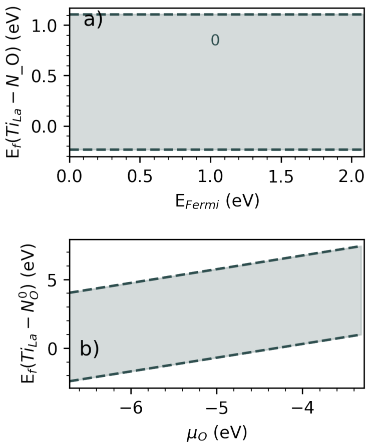

FIG. S62: a) $Ti_{La}-N_O$ formation energy ($E_f(Ti_{La})N_O$) in the neutral charge state computed as a function of the Fermi energy (E_{Fermi}, up to the experimental band gap E_g^{expt}) referenced to the valence band maximum of LTON and for $\Delta\mu_O$=-4.54 eV. b) Formation energy for a $(Ti_{La}-N_O)^0$ as a function of the oxygen chemical potential and for $E_{Fermi}= E_g^{expt}$. The colored area indicates the variation of $E_f(Ti_{La}N_O)$ for different configurations.

TABLE S22: Formation energy (in eV) for a neutral $(Ti_{La}-N_O)^0$ in LTON and distance between the two defects ($d(Ti_{La}-N_O)$)

Conf.	$d(Ti_{La}-N_O)$	$Ti_{La}-N_O{}^0$
1	2.27	0.30
2	2.65	0.61
3	4.25	0.81
4	5.42	0.75
5	4.82	0.91
6	4.77	0.91
7	1.96	-0.08
8	1.98	-0.08
9	2.56	0.59
10	5.12	1.10
11	4.61	0.60
12	2.02	-0.24
13	4.34	0.60
14	2.61	-0.24
15	2.97	0.59
16	4.80	1.10

S6. EFFECT OF THE N CHEMICAL POTENTIAL

Figures S63 and S64 summarize the formation energies of all considered defects in LTO and LTON respectively, considering either N_2 or NH_3 as reference for the N chemical potential. For LTON, we additionally consider Ti-rich/La-poor and Ti-poor/La-rich conditions. In our calculations, $\mu_N^0(NH_3)$ is found to be 1.01 eV higher than $\mu_N^0(N_2)$ and consequently when NH_3 is used as a reference, N-related defects are slightly destabilized. In LTO, the relative stability of N-related defects with the same N content is not affected, but defects with a larger N content (N_i-N_O and N_O-N_O) are more strongly affected and become more stable than single N-based defects for lower values of the O chemical potential compared to using N_2 as reference. Similarly in LTON, the relative stability of N-based defects is not affected, but these defects are destabilized with respect to O-based defects, especially for O-rich conditions.

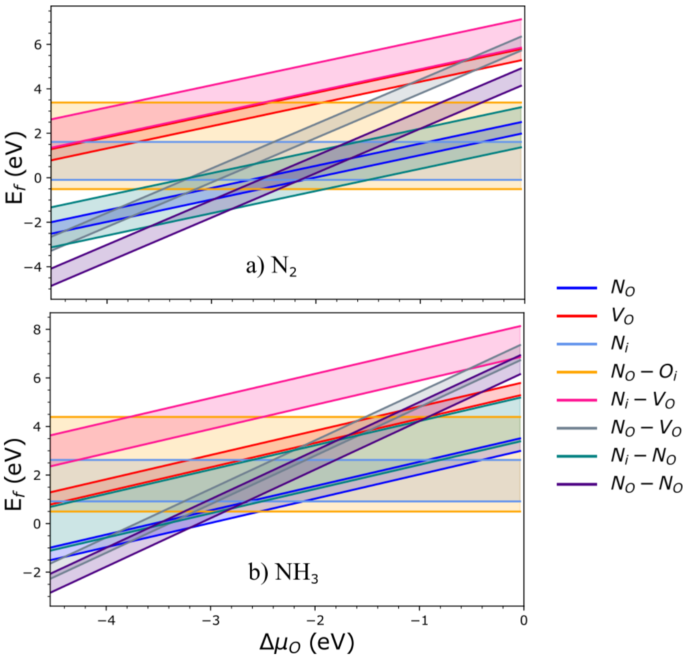

FIG. S63: Evolution of the formation energy in the experimentally relevant charge state ($E_{\mathrm{Fermi}} = E_g^{\mathrm{expt}}$) of the considered defects in LTO as a function of the oxygen chemical potential from O-rich ($\Delta\mu_O = 0$ eV) to O-poor ($\Delta\mu_O = -4.54$ eV) conditions. For each defect, the colored area indicates the variation of E_f for different configurations. In panels a) and b) the formation energy is computed using N_2 and NH_3 as reference of the N chemical potential, respectively.

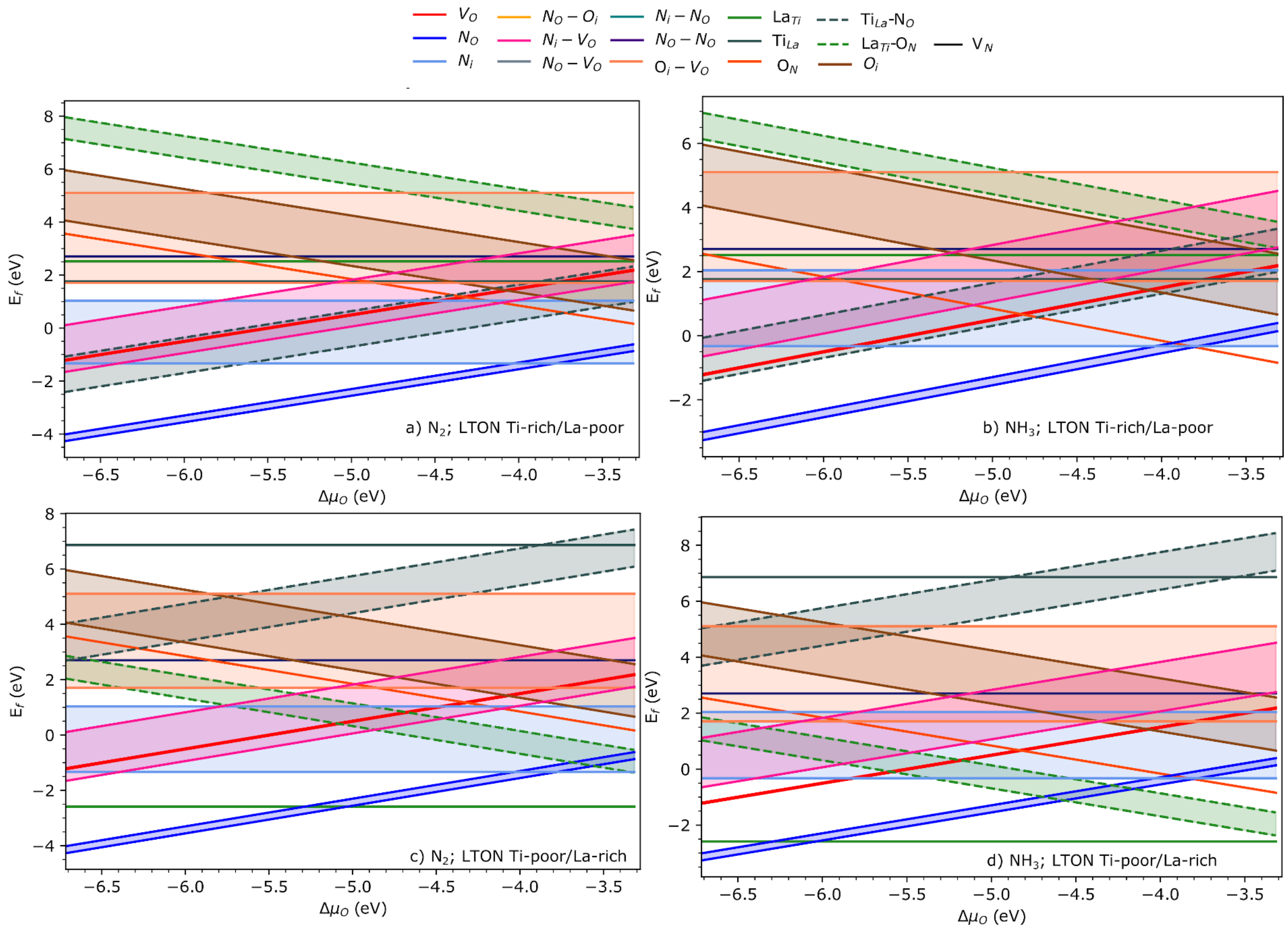

FIG. S64: Evolution of the formation energy in the experimentally relevant charge state ($E_{\mathrm{Fermi}} = E_g^{\mathrm{expt}}$) of the considered defects in LTON as a function of the oxygen chemical potential from O-rich ($\Delta\mu_{\mathrm{O}} = -3.28$ eV) to O-poor ($\Delta\mu_{\mathrm{O}} = -6.72$ eV) conditions. For each defect, the colored area indicates the variation of E_{f} for different configurations. In panels a) and c) the formation energy is computed using N_2 as a reference for the N chemical potential, while for panels b) and d) NH_3 was used.

[1] H. W. Schmalle, T. Williams, A. Reller, A. Linden, and J. G. Bednorz, Acta. Crystallogr. B **49**, 235 (1993).
[2] H. G. Kim, D. W. Hwang, J. Kim, Y. G. Kim, and J. S. Lee, Chem. Commun. **12**, 1077 (1999).
[3] D. W. Hwang, J. S. Lee, W. Li, and S. H. Oh, J. Phys. Chem. B **107**, 4963 (2003).
[4] D. W. Hwang, H. G. Kim, J. S. Lee, J. Kim, W. Li, and S. H. Oh, J. Phys. Chem. B **109**, 2093 (2005).
[5] S. Ninova and U. Aschauer, J. Mater. Chem. A **5**, 11040 (2017).
[6] A. Jain, G. Hautier, S. P. Ong, C. J. Moore, C. C. Fischer, K. A. Persson, and G. Ceder, Phys. Rev. B **84**, 045115 (2011).
[7] F. Tessier, L. Le Gendre, F. Cheviré, R. Marchand, and A. Navrotsky, Chem. Mater. **17**, 3570 (2005).
[8] P. Liu, J. Nisar, B. Sa, B. Pathak, and R. Ahuja, J. Phys. Chem. C **117**, 13845 (2013).
[9] J. Zhang, W. Dang, Z. Ao, S. K. Cushing, and N. Wu, Phys. Chem. Chem. Phys. **17**, 8994 (2015).
[10] Z. Ma, K. Wu, R. Sa, Q. Li, C. He, and Z. Yi, Int. J. Hydrog. **40**, 980 (2015).
[11] F. Meng, Z. Hong, J. Arndt, M. Li, M. Zhi, F. Yang, and N. Wu, Nano Res. **5**, 213 (2012).
[12] A. Fujishima, X. Zhang, and D. A. Tryk, Surf. Sci. Rep. **63**, 515 (2008).
